# Bioinspired Hierarchical Hydrogel Electrolyte for Ultralong-Life Flexible Zinc-Ion Batteries

**DOI:** 10.1007/s40820-026-02246-0

**Published:** 2026-06-10

**Authors:** Ran Wang, Qian Gao, Runhai Wu, Yongqi Mi, Shaopei Yang, Hongxiao Wang, Ting Wan, Sehrish Gull, Kefeng Xie, Guankui Long, Pengcheng Du

**Affiliations:** 1 State Key Laboratory of Natural Product Chemistry and Institute of Polymer Science and Engineering, College of Chemistry and Chemical Engineering, Lanzhou University, Lanzhou, 730000 People’s Republic of China; 2 Frontiers Science Center for New Organic Matter, Tianjin Key Lab for Rare Earth Materials and Applications, Renewable Energy Conversion and Storage Center (RECAST), School of Materials Science and Engineering, Nankai University, Tianjin, 300350 People’s Republic of China; 3 School of Chemistry and Chemical Engineering, Lanzhou Jiaotong University, Lanzhou, 730070 People’s Republic of China

**Keywords:** Zinc-ion battery, Hydrogel electrolyte, MXene, Hierarchical structure, Zn^2+^ rapid transport channel

## Abstract

Hydrogel electrolytes are pivotal for flexible zinc-ion batteries (ZIBs) yet suffer from an intrinsic trade-off between mechanical robustness and ionic conductivity. Herein, drawing inspiration from the “adhesion-conduction” architecture of spider webs, we developed a hierarchical hydrogel electrolyte (MTP) by incorporating tannic acid (TA)-modified MXene nanosheets (MT) into a polyacrylamide (PAM) skeleton to construct uniform 3D ion-conductive pathways. This bioinspired hierarchy serves a dual function: The PAM framework ensures mechanical integrity, while the MT network creates directed low-resistance channels for Zn^2+^ transport. Specifically, the dense array of polar groups on MXene and phenolic hydroxyls on TA act as “sticky sites”, which accelerate desolvation kinetics and homogenize Zn^2+^ flux. Consequently, the MTP electrolyte achieves an impressive ionic conductivity of 27.69 mS cm^−1^ and a high Zn^2+^ transference number of 0.833. Enabled by this design, Zn//Zn symmetric cells demonstrate an ultralong lifespan of 4600 h (> 6 months) at 0.5 mA cm^−2^/0.5 mAh cm^−2^. Furthermore, Zn//Z-VO full cells exhibit outstanding cyclability, retaining 74.5% capacity after 2000 cycles at 2 A g^−1^ and maintaining durable operation for over 10,000 cycles at 5 A g^−1^. This work successfully translates a biological blueprint into a practical strategy for resolving the kinetic and stability challenges in high-performance flexible ZIBs.

**Supplementary Information:**

The online version contains supplementary material available at 10.1007/s40820-026-02246-0.

## Highlights

- Drawing inspiration from the “adhesion-conduction” architecture of spider webs, a hierarchical hydrogel electrolyte (MTP) is developed with uniform 3D ion-conductive pathways.
- Specific “sticky sites” on the MXene/tannic acid network facilitate superior electrochemical properties, including a high Zn^2+^ transference number of 0.833 and ionic conductivity of 27.69 mS cm^−1^.
- This strategy enables stable Zn anodes with an impressive lifespan of 4600 h (>6 months) and empowers flexible full cells with outstanding cyclability over 10,000 cycles.

**Supplementary Information:**

The online version contains supplementary material available at 10.1007/s40820-026-02246-0.

## Graphical abstract

**Figure:**
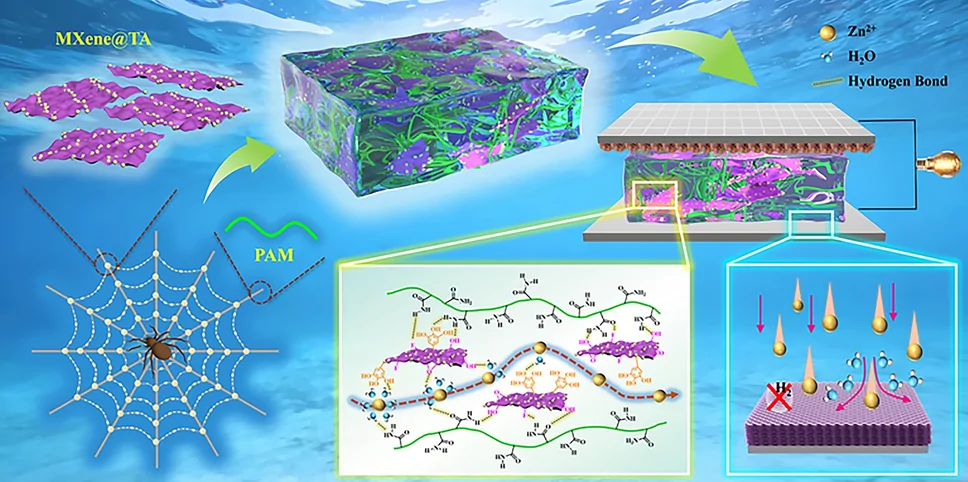

## Introduction

The energy-intensive nature of traditional industrial processes, coupled with their significant carbon footprint, has exacerbated escalating environmental crises such as global warming and pollution, making the transition from fossil fuels to sustainable renewable energy an urgent global imperative [1, 2]. Within the new energy system, advanced energy storage systems emerge as a pivotal component, addressing the intermittent challenges of renewable energy sources [3]. While lithium-ion batteries (LIBs) currently dominate the market, their application is increasingly constrained by safety hazards, resource scarcity, and recycling complexities [4]. Consequently, aqueous zinc-ion batteries (ZIBs) have emerged as compelling candidates for the post-lithium era, distinguished by their high theoretical capacity (820 mAh g^−1^), low redox potential (− 0.76 V *vs.* SHE), and intrinsic safety [5–7].

Despite the aforementioned advantages, the practical commercialization of ZIBs is impeded by two persistent and interrelated challenges: interfacial side reactions and dendrite growth [8, 9]. In conventional aqueous electrolytes, the thermodynamic instability of hydrated Zn^2+^ triggers the reductive decomposition of water. This leads to localized electrolyte alkalization and the formation of insulating by-products (e.g., Zn_4_SO_4_(OH)_6_·3H_2_O), which deposit on the anode surface, thereby increasing interfacial impedance and hindering ion transport [10]. Furthermore, the uneven accumulation of these by-products creates heterogeneous nucleation sites that exacerbate the “tip effect”, ultimately inducing disordered dendrite growth and catastrophic cell failure. These fundamental challenges continue to hinder the further commercialization of ZIBs [11, 12]. To address these issues, optimizing the electrolyte composition is considered a pivotal strategy for extending the lifespan of ZIBs. Current research focuses on diverse strategies, including aqueous electrolyte additives [13, 14], gel polymer electrolytes [15, 16], and all-solid-state electrolytes [17, 18]. However, aqueous electrolytes offer fast diffusion kinetics but suffer from severe water-induced parasitic reactions [19], whereas solid-state electrolytes effectively suppress zinc dendrites but generally exhibit poor ionic conductivity [20, 21].

Hydrogel electrolytes have emerged as a pivotal solution for flexible electronics, effectively bridging the gap between the high ionic conductivity of aqueous electrolytes and the mechanical integrity of solid-state counterparts [22]. To date, various polymer matrices have been extensively developed for ZIBs, such as polyacrylamide (PAM), polyvinyl alcohol (PVA), sodium alginate (SA), carboxymethyl cellulose (CMC), or chitosan (CS) [23–27]. These polymers typically feature abundant polar functional groups (e.g., –COOH, –OH, –NH_2_) that can reinforce the hydrogel network via hydrogen bonding to enhance mechanical properties and coordinate with water molecules to regulate the Zn^2+^ solvation structure [26]. However, an intrinsic trade-off persists in these systems. The strong interactions required to restrict free water activity and suppress interfacial side reactions often inadvertently immobilize Zn^2+^, resulting in sluggish transport kinetics and severe concentration polarization at high current densities compared to aqueous electrolytes [28, 29]. Consequently, developing a hydrogel architecture that simultaneously achieves robust water confinement and rapid ion transport remains a significant challenge.

Nature often provides elegant solutions to complex engineering challenges. Biological hydrogels, in particular, have inspired the development of electrolyte architectures with optimized functionalities [30, 31]. For instance, mimicking the gradient structure of articular cartilage, Wang et al. designed an asymmetric hydrogel network (PCG20-PC5) that modulates crosslinking density to balance ionic conductivity with water suppression, achieving stable cycling for over 2200 h under 1 mA cm^−2^/1 mAh cm^−2^ [32]. Beyond gradient designs, the spider web represents a masterpiece of hierarchical engineering, relying on a synergistic “capture-conduction” mechanism. Its architecture comprises a radial thread framework that ensures mechanical integrity and rapid signal transmission, integrated with viscid droplets rich in hydrophilic groups. These droplets function as “sticky traps” to capture prey via robust molecular interactions, while the interconnected threads facilitate rapid response [33]. This hierarchical “adhesion-conduction” synergy offers a compelling blueprint for designing next-generation hydrogel electrolytes that require both precise ion confinement and fast transport kinetics.

Drawing cues from the “adhesion-conduction” mechanism of spider webs, we engineered a hierarchical hydrogel electrolyte (MTP) to enable fast and stable Zn^2+^ transport. The hydrogel comprises a PAM skeleton mimicking the web’s radial threads, reinforced with MXene@TA (MT) nanocomposites that act as “sticky capture traps”. The abundant polar groups on MT facilitate the desolvation of Zn^2+^, separating ions from free water, thereby creating selective channels for rapid Zn^2+^ transport. This design simultaneously enhances Zn^2+^ transport kinetics (27.69 mS cm^−1^ of ionic conductivity, $t_{{Zn^{2 + } }} \,$= 0.833). As a result, the MTP electrolyte enables Zn//Zn symmetric batteries to achieve an ultralong cycling life of 4600 h at 0.5 mA cm^−2^/0.5 mAh cm^−2^. Notably, the system proves its practical viability through Zn//Z-VO full batteries (> 2000 cycles at 2 A g^−1^) and high-capacity pouch cells (234.22 mAh g^−1^). This bioinspired strategy offers a robust solution for constructing next-generation flexible ZIBs.

## Experimental Section

### Materials

Titanium aluminum carbide (Ti_3_AlC_2_, 98%) was obtained from Jilin 11 Technology Co., Ltd. (China). Lithium fluoride (LiF, 98%) was provided by Heowns Biochem Technologies Co., Ltd. (China). Hydrochloric acid (HCl, 36.0–38.0%) was purchased from Xilong Scientific Co., Ltd. (China). Tannic acid (TA), ammonium persulfate (APS, 98%) and 1-methyl-2-pyrrolidinone (NMP, 99%) were supplied by Shanghai Macklin Biochemical Co., Ltd. (China). Acrylamide (AM, 99.0%), N,N’-methylenebisacrylamide (MBAA, 99%), zinc sulfate heptahydrate (ZnSO_4_∙7H_2_O, 99%) and vanadium pentoxide (V_2_O_5_, 99%) were obtained from Shanghai Aladdin Biochemical Technology Co., Ltd. (China). Hydrogen peroxide (H_2_O_2_, 30 wt.%) was obtained from Tianjin Damao Chemical Reagent Factory (China). Acetylene black (99%) was obtained from Alfa Aesar (USA). Polyvinylidene fluoride (PVDF, M_w _≈ 8 × 10^6^) was obtained from Arkema (France). All chemicals were used as received without further purification. Deionized (DI) water was used throughout the experiment.

### Fabrication of MXene Nanosheets and MXene@TA Nanocomposites [34]

Ti_3_AlC_2_ MXene nanosheets were synthesized via a selective etching method. Briefly, 1.6 g of LiF was dissolved in 20 mL of 9 M HCl under stirring in an ice bath. Subsequently, 1.0 g of Ti_3_AlC_2_ powder was slowly added and etched at 40 °C for 48 h. The resulting mixture was centrifuged to collect the sediment, which was then washed repeatedly with 0.1 M HCl and DI water until the supernatant pH exceeded 6. The purified sediment was re-dispersed in DI water, purged with Ar gas, and ultrasonicated for 2 h. The suspension was then centrifuged to remove unexfoliated particles, and the supernatant was freeze-dried to obtain few-layer MXene nanosheets.

To prepare MXene@TA (MT) nanocomposites, MXene nanosheets were dispersed in DI water, followed by the addition of an equal mass of TA. The mixture was stirred continuously for 12 h to ensure self-assembly.

### Synthesis of MXene@TA-PAM (MTP), MXene-PAM (MP), TA-PAM (TP) and Pure PAM Hydrogel

The MTP hydrogel was synthesized via a facile one-pot thermal polymerization method. ZnSO_4_·7H_2_O was dissolved in 8 mL of the as-prepared MT dispersion to form a 2 M ZnSO_4_ electrolyte. Subsequently, 1.2 g of AM, 4 mg of MBA and 40 mg of APS were sequentially added and stirred until dissolved. The precursor solution was degassed, poured into a glass mold and polymerized at 60 °C for 2 h. To optimize the composition, hydrogels with varying MXene contents (10, 24, 50, 80 and 100 mg) were prepared and designated as MTP-1, MTP-2, MTP-3, MTP-4 and MTP-5, respectively.

For comparison, pure PAM, MP and TP hydrogels were synthesized following the same procedure, substituting the MT dispersion with DI water, the pure MXene dispersion or the pure TA solution, respectively.

### Preparation for Z-VO [35]

Zn^2+^-pre-intercalated V_2_O_5_ (Z-VO) was synthesized via a hydrothermal method. First, 0.364 g of V_2_O_5_ powder was dispersed in 30 mL of DI water. 2 mL of H_2_O_2_ (30 wt%) was added dropwise, and the mixture was stirred at room temperature for 1 h to form a transparent orange peroxovanadate solution. Then, 0.75 mmol of ZnSO_4_ was added and stirred for another hour. The homogeneous solution was transferred to a 100-mL Teflon-lined stainless steel autoclave and heated at 120 °C for 24 h. After cooling to room temperature, the precipitate was collected by centrifugation, washed three times with DI water and ethanol and dried at 60 °C to obtain Z-VO powder.

To fabricate the cathode, Z-VO active material, acetylene black and PVDF were mixed in a weight ratio of 7:2:1 using NMP as the solvent. The resulting slurry was cast onto a Ti foil current collector and vacuum-dried at 60 °C for about 12 h. The active mass loading was approximately 3.0 mg cm^−2^.

## Results and Discussion

### Design and Characterization of Hydrogel Electrolytes

**Fig. 1:**
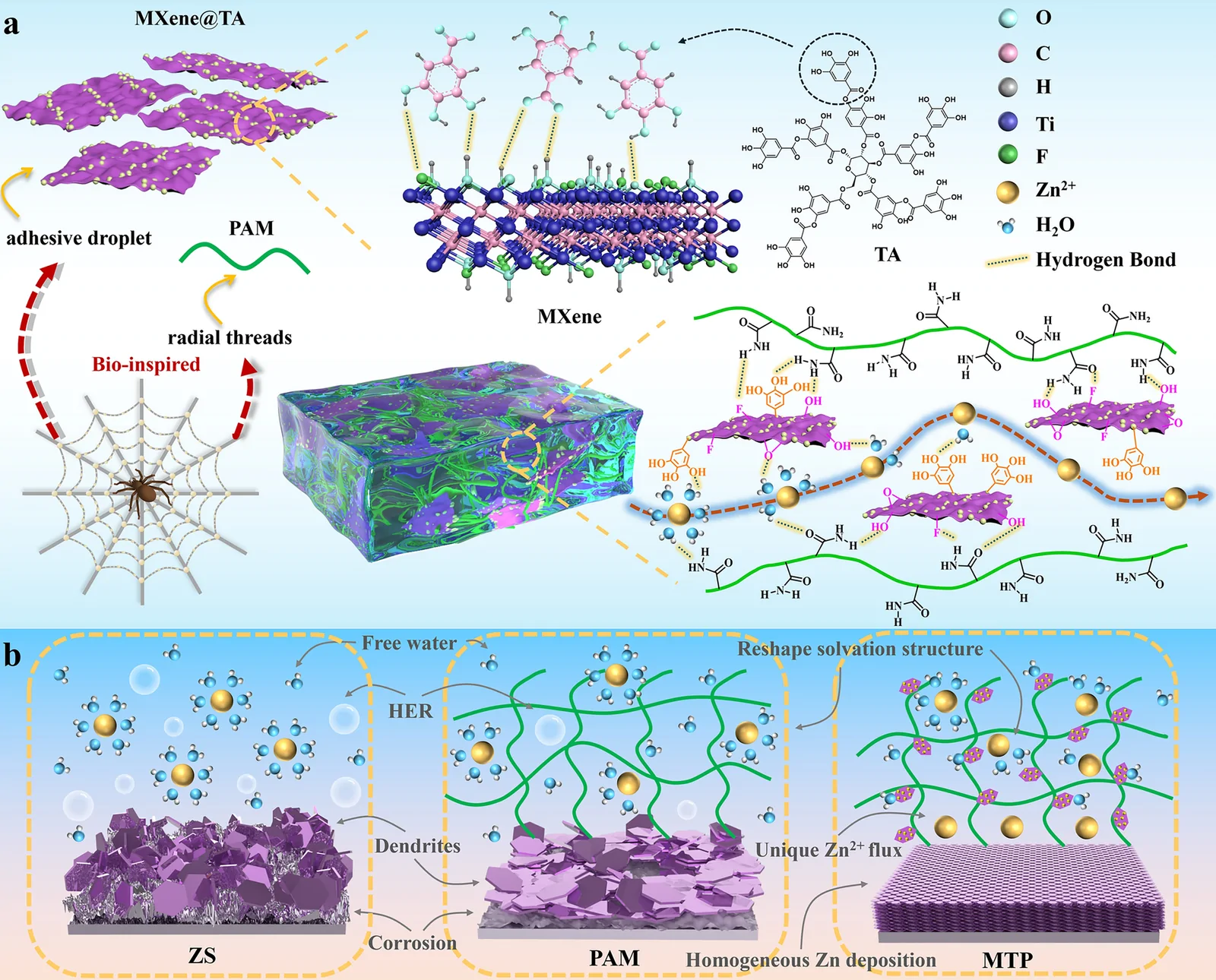
**a** Schematic diagram of a spider-web-inspired hierarchical structured hydrogel. **b** Schematic illustrations of Zn anodes in ZS, PAM hydrogel and MTP hydrogel electrolytes

Inspired by the “adhesion-conduction” architecture of spider webs, we engineered a hierarchical conductive hydrogel electrolyte (MTP) featuring directed channels for rapid Zn^2+^ transport (Fig. 1a). In this bioinspired design, the PAM matrix functions as the robust “radial thread” skeleton, establishing a 3D network. Embedded within this matrix are MT nanocomposites, formed via the self-assembly of TA onto MXene nanosheets, which serve as the functional “sticky droplets”. The schematic in Fig. 1b illustrates the fundamental challenges addressed by this design. In conventional aqueous electrolytes (ZS), the abundance of free water inevitably triggers uncontrolled zinc dendrite growth and severe parasitic reactions. The resulting hydrogen evolution reaction (HER) induces local pH fluctuations and accumulates internal pressure, severely compromising battery stability [36–38]. Conventional hydrogel electrolytes mitigate these issues by immobilizing water via hydrophilic polymer chains. However, this often introduces new limitations: Their quasi-solid nature leads to poor interfacial contact, and the strong restriction of solvent activity typically comes at the cost of sluggish Zn^2+^ transport kinetics. This results in severe concentration polarization, degrading Coulombic efficiency (CE) and cycle life [39]. In contrast, the MTP electrolyte resolves these trade-offs through a synergistic mechanism. The abundant negatively charged surface groups of MXene, combined with the phenolic hydroxyls of TA, construct a dense array of Zn^2+^ adsorption sites. These sites effectively coordinate with water molecules to suppress HER and other side reactions. Crucially, unlike traditional hydrogels, these functional groups create low-energy-barrier transport channels that significantly enhance Zn^2+^ transport kinetics. This architecture effectively homogenizes Zn^2+^ flux and guides uniform nucleation, establishing a highly oriented, dendrite-free deposition interface [40].

**Fig. 2:**
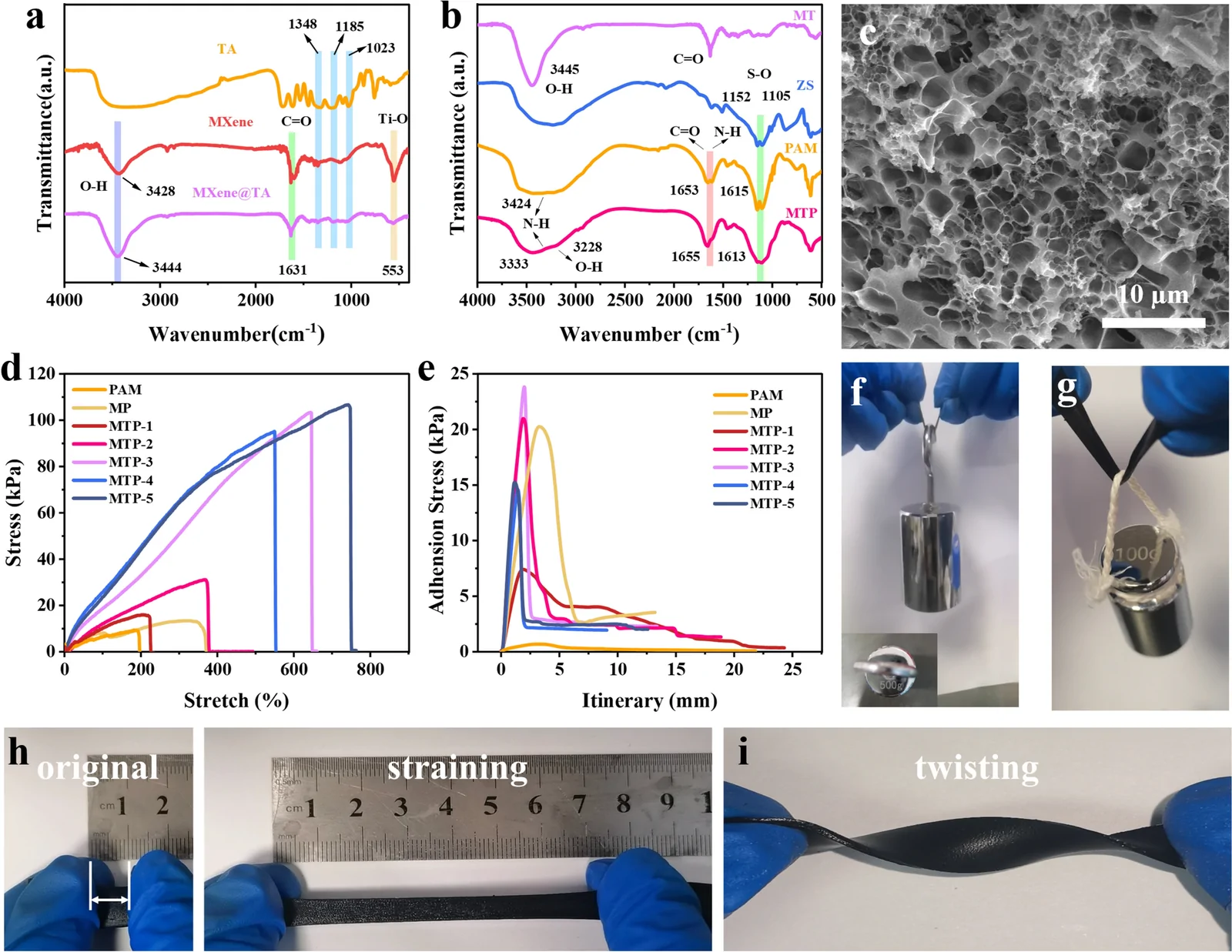
FTIR spectra of **a** TA, MXene and MT and **b** MT, ZS, PAM and MTP hydrogel. **c** SEM image of freeze-dried MTP gel. **d** Stress–strain curves. **e** Adhesion curves. Photograph of **f** MTP-5 lifting a 500 g weight, **g** MTP-2 lifting a 100 g weight, **h** original (left) and straining (right) state of MTP-5 and **i** twisting stability of the MTP-5

MXene nanosheets were prepared by selectively etching the Al layer from Ti_3_AlC_2_ using a LiF/HCl mixture. The resulting MXene exhibited a classic accordion-like morphology with stacked layers, revealing the 2D nanosheet structure (Fig. S1a, b). X-ray diffraction (XRD) patterns further confirmed the successful synthesis of the MXene (Fig. S1c). However, pristine MXene is susceptible to oxidation by dissolved oxygen in aqueous environments, leading to degradation and loss of conductivity [40, 41]. To address this, TA was introduced to self-assemble onto the MXene surface via hydrogen bonding. The TA layer functions as a sacrificial antioxidant, where its phenolic hydroxyl groups are preferentially oxidized, thereby protecting the intrinsic Ti–C bonds of MXene [42, 43]. The synthesized MT nanocomposites retained a distinct sheet-like morphology, as evidenced by scanning electron microscopy (SEM) and transmission electron microscopy (TEM) (Fig. S2a, b). XRD analysis revealed a shift in diffraction peaks, confirming the intercalation of TA molecules between MXene layers (Fig. S2c). X-ray photoelectron spectroscopy (XPS) was employed to evaluate the oxidation resistance of MT (Fig. S3). The high-resolution Ti 2*p* spectrum indicated a reduced proportion of TiO_2_ in MT (9.34%) compared to pristine MXene (12.11%) (Fig. S4a). Due to Ti’s high reactivity, water molecules or dissolved oxygen in the air attacks the Ti–C bonds, causing oxygen-containing groups to substitute the lattice carbon. As Ti and O continuously enrich in nanoscale regions, TiO_2_ eventually nucleates and grows, ultimately destroying the 2D layered structure of MXene. Therefore, the decreased TiO_2_ content in MT confirms the enhanced oxidation stability of the composite [44]. The chemical interaction between MXene and TA was further elucidated by Fourier transform infrared spectroscopy (FTIR) (Fig. 2a). Pristine MXene displayed characteristic peaks at 3428, 1631 and 553 cm^−1^, corresponding to –OH, C = O and Ti–O vibrations, respectively [45]. In the MT spectrum, the O–H stretching vibration shifted to 3444 cm^−1^, suggesting the formation of intermolecular hydrogen bonds. Additionally, new peaks appeared at 1348, 1185 and 1023 cm^−1^, attributed to the C–O stretching, O–H bending and C–H bending vibrations of TA [46, 47]. Collectively, these results confirm the successful synthesis of MT nanocomposites with enhanced oxidation stability.

MTP hydrogels were synthesized via a facile one-pot polymerization method by dispersing MT into a PAM precursor solution containing 2 M ZnSO_4_. The chemical structure of the MTP hydrogel was analyzed by FTIR (Fig. 2b). Characteristic peaks at 1152 and 1105 cm^−1^ were assigned to the SO^2-^_4_ groups from ZnSO_4_ [48]. Notably, the N–H stretching vibration of PAM (3424 cm^−1^) and the O–H vibration of MT (3240 cm^−1^) shifted to 3333 and 3228 cm^−1^ in the MTP spectrum, respectively. These shifts indicate the formation of a robust hydrogen-bond network between the MT filler and the PAM matrix. Furthermore, peaks at 1655 and 1613 cm^−1^ correspond to the amide groups (–CONH_2_) of PAM, with the C = O vibration intensity enhanced by the contribution from MT [49].

To optimize the hydrogel composition, a series of MTP samples with varying MT contents (MTP-1 to MTP-5) were prepared. Control samples included pure PAM, MP and TP. It is noteworthy that the TP precursor failed to fully polymerize, remaining partially fluid (Fig. S5). This is likely because excessive TA acts as a radical scavenger, terminating the chain propagation during polymerization [50]. However, in the MTP system, the adsorption of TA onto MXene mitigates this inhibitory effect. Morphologically, the MTP-2 hydrogel exhibits a uniform, interconnected 3D porous network (Fig. 2c). This architecture is critical for the bioinspired “conduction” function, facilitating rapid ion transport while providing a stable structural scaffold. In contrast, the larger and more disordered pores of the pristine PAM hydrogel fail to provide the continuous pathways required for fast ion transport and lack the structural robustness needed for stable electrode–electrolyte contact. Conversely, excessive MT loading triggers local phase separation and filler aggregation, generating interfacial voids between the filler and the polymer matrix that undesirably enlarge the porous structure (Fig. S6). Such oversized and irregular pores increase the tortuosity of ion transport pathways, exacerbate concentration polarization and consequently accelerate dendrite growth. Mechanical testing revealed that the incorporation of MT significantly enhances the mechanical performance of the hydrogels (Fig. 2d). Both tensile strength and elongation at break increased with higher MT content. The optimized MTP-5 hydrogel achieved a tensile strength of 106.64 kPa and an exceptional elongation of 763% (Fig. 2h). This enhancement is attributed to the 2D layered structure of MXene, which enables interlayer sliding and effectively dissipates energy under stress. Moreover, the strong covalent bonds (Ti–C, Ti–N) within MXene enhance the intrinsic toughness of the matrix [51, 52]. However, adhesion performance declined when MXene content exceeded 0.05 g (MTP-4 and MTP-5) (Fig. 2e). This reduction is likely due to the hydrogel’s increased stiffness at high filler loadings, which compromises its ability to conform to the substrate surface. Furthermore, the MTP hydrogels demonstrated excellent flexibility, withstanding twisting and bending without fracture (Fig. 2i). These superior mechanical and adhesive properties suggest that MTP hydrogels are well-suited for flexible wearable energy storage devices. In addition, the water retention property of the hydrogel was evaluated (Fig. S7); the MTP hydrogel maintained a water retention rate of 70.88% after 4 days, significantly outperforming the PAM hydrogel (50.58%). This enhanced performance is attributed to the abundant polar groups within the MT composite, which exhibit a considerable capacity for binding water molecules, thereby inhibiting water evaporation from the hydrogel. This further validates the advantage of MTP in maintaining mechanical stability for practical applications.

### Enhancement Mechanism of MTP Hydrogel Electrolytes

**Fig. 3:**
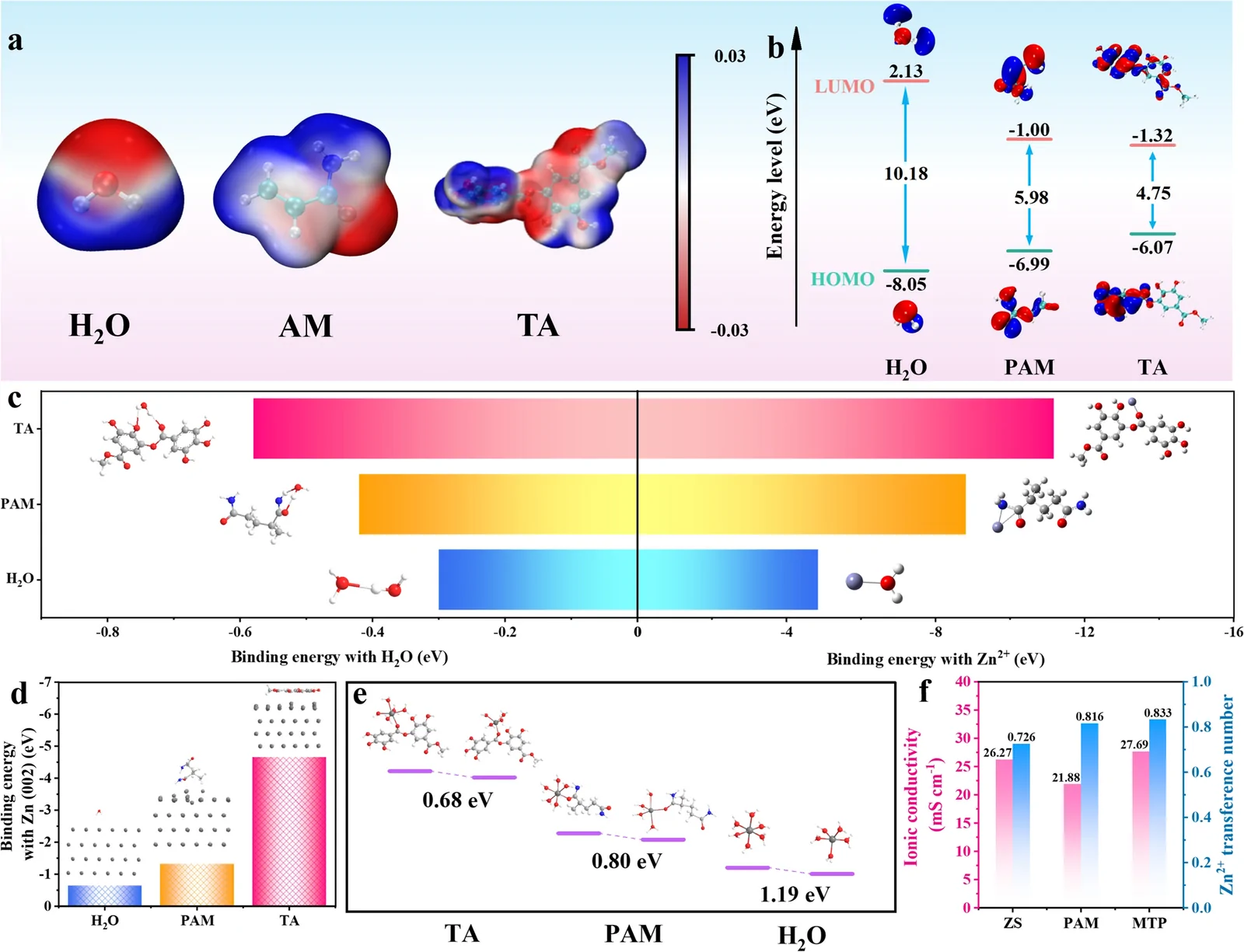
**a** ESP of H_2_O, AM and TA molecules. **b** HOMO–LUMO energy-level distribution of H_2_O, AM and TA molecules. **c** Binding energy of H_2_O, AM and TA molecules with H_2_O and Zn^2+^. **d** Binding energy of H_2_O, AM and TA molecules with Zn (002). **e** Desolvation energy of H_2_O, AM and TA molecules. **f** Ionic conductivity and Zn^2+^ transference number of diverse electrolytes

Density functional theory (DFT) calculations were performed to elucidate the regulatory mechanism of electrolyte components at the molecular level. Molecular electrostatic potential (ESP) mapping (Fig. 3a) reveals that TA possesses broad and intense negative potential regions around its phenolic hydroxyl oxygens. This electronic characteristic identifies TA as a molecule rich in zincophilic active sites, providing electronic-scale evidence for its preferential adsorption and interfacial regulation function [53]. Additionally, frontier orbital analysis (Fig. 3b) shows that TA exhibits the narrowest highest occupied molecular orbital–lowest unoccupied molecular orbital (HOMO–LUMO) bandgap (4.75 eV) compared to PAM and H_2_O, suggesting superior electron transfer capability and the potential for uniform electric field modulation [54].

In terms of maintaining interface stability, TA exhibits a synergistic mechanism involving hydrogen bonding and coordination interactions. As shown in Fig. 3c, TA exhibits a significantly higher binding energy with H_2_O (− 0.58 eV) compared to PAM (− 0.42 eV) and H_2_O–H_2_O interactions (− 0.30 eV). This strong affinity, attributed to the dense network of phenolic hydroxyl groups, effectively “locks” free water molecules via hydrogen bonding, thereby reducing water activity and mitigating the HER. Furthermore, TA indicates a preferential adsorption on the Zn (002) surface with a binding energy of − 4.66 eV, far surpassing that of H_2_O (− 0.64 eV) and PAM (− 1.32 eV) (Fig. 3d). This robust adsorption forms a protective layer that physically excludes water from the anode surface, further inhibiting corrosion [55].

Crucially, TA modulates the Zn^2+^ solvation structure and enhances deposition kinetics. Due to its strong capability of binding with Zn^2+^ (Fig. 3c), TA is able to act as the primary coordination site within the solvation sheath and achieve efficient selective capture of Zn^2+^. Thereby, it accomplishes the initial-stage “adhesion” function within the biomimetic hierarchical system. The resulting TA-coordinated complex exhibits a narrowed HOMO–LUMO gap (Fig. S8), which facilitates interfacial charge transfer [56]. More importantly, desolvation energy calculations (Fig. 3e) reveal that stripping a water molecule from the [Zn(TA)(H_2_O)_5_]^2+^ complex requires only 0.68 eV. This barrier is drastically lower than that of the hydrated [Zn(H_2_O)_6_]^2+^ cation (1.19 eV) or the [Zn(PAM)(H_2_O)_5_]^2+^ complex (0.80 eV). This significantly reduced energy barrier accelerates the desolvation process, thereby preventing the sluggish kinetics that typically induce the “tip effect”. Therefore, the TA molecule suppresses interfacial side reactions and electrode corrosion by binding with water molecules and the Zn anode surface, guides the uniform nucleation of Zn^2+^ at the electrode surface through optimized desolvation kinetics and consequently inhibits dendrite growth, thereby maintaining the long-term stability of the Zn anode.

### Stability Evaluation of Zn Anode

To identify the optimal hydrogel composition, Zn//Zn symmetric cells were assembled to evaluate the stability testing. As shown in Fig. S9, the MTP-2 hydrogel demonstrated the most robust cycling performance (800 h) under harsh conditions (4 mA cm^−2^, 4 mAh cm^−2^). In addition, MTP-2 exhibits the highest ionic conductivity among MTP series hydrogels (Fig. S10). Rapid ion transport kinetics facilitate uniform ion flux and promote homogeneous deposition. Overall, hydrogels with superior mechanical properties sacrifice their adhesion to the substrate surface due to increased stiffness, which compromises the actual interface stability of the battery. In contrast, MTP-2 achieves an optimal balance among mechanical enhancement, interfacial adhesion and electrochemical stability. Therefore, MTP-2 was selected for all subsequent evaluations (denoted as MTP). Generally, hydrogel electrolytes suffer from inferior ionic conductivity compared to aqueous solutions due to restricted polymer chain mobility and reduced free water content [57]. However, the hierarchical MTP architecture effectively overcomes this limitation. Electrochemical impedance spectroscopy (EIS) on Ti//Ti cells revealed that the MTP electrolyte achieves a remarkable ionic conductivity of 27.69 mS cm^−1^, surpassing both the pure PAM hydrogel (21.88 mS cm^−1^) and the aqueous ZS electrolyte (26.27 mS cm^−1^) (Figs. 3f and S11). Furthermore, the Zn^2+^ transference number ($t_{{{\mathrm{Zn}}^{2 + } }}$) of the MTP hydrogel, evaluated via the Bruce–Vincent method, was calculated to be 0.833. This value significantly exceeds those of PAM (0.816) and ZS (0.726) (Figs. 3f and S12) and notably outperforms most recently reported hydrogel electrolytes (Table S1). This enhanced transport kinetics is attributed to a threefold synergistic mechanism: (1) The 2D layered structure of MXene provides low-barrier pathways for ion intercalation; (2) the dense array of anionic groups (-F, -O) on MXene and phenolic hydroxyls on TA serve as “sticky sites”, facilitating rapid Zn^2+^ migration; and (3) the interconnected porous network minimizes the tortuosity of diffusion channels.

**Fig. 4:**
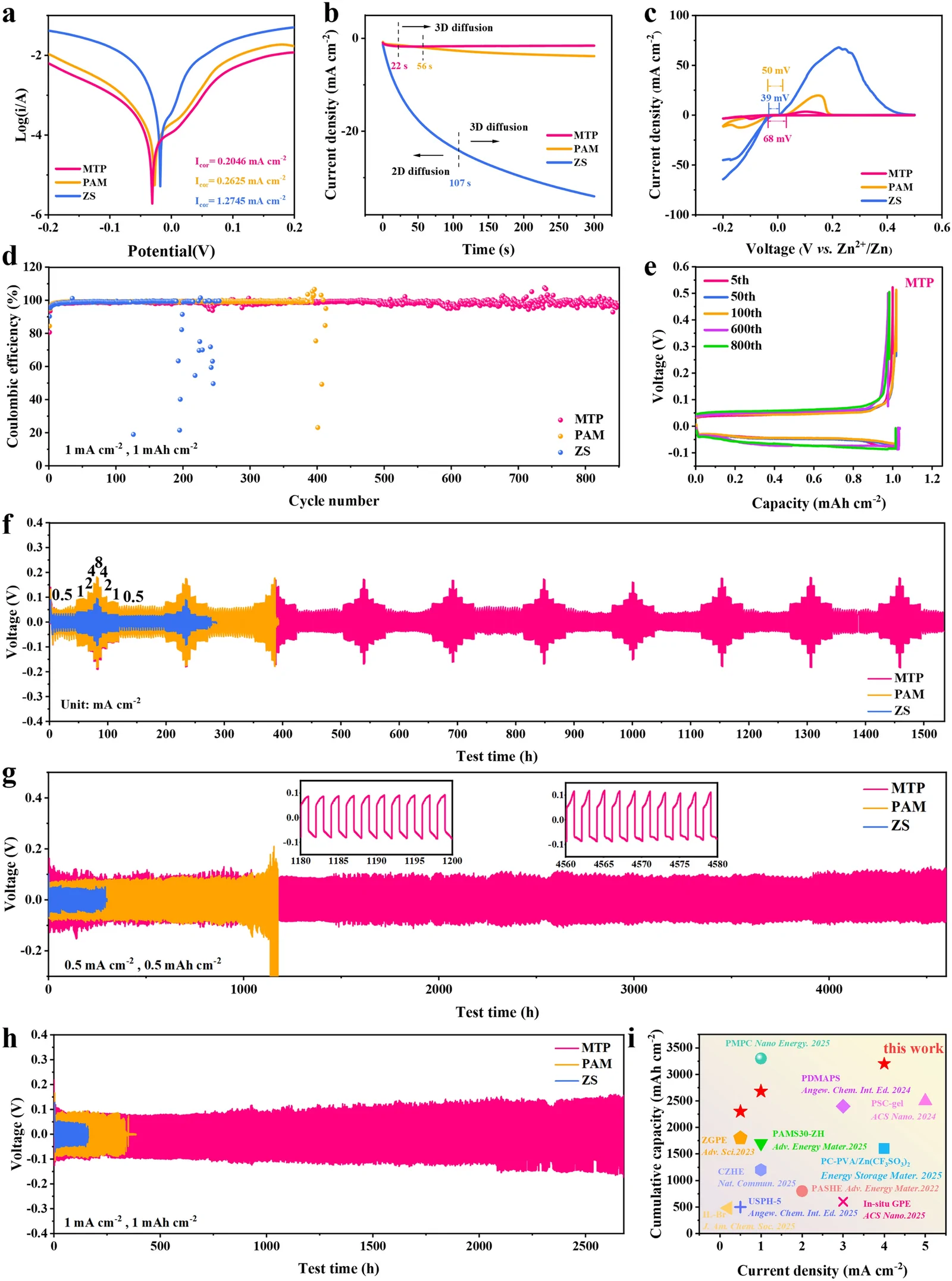
**a** Tafel curves. **b** CA tests. **c** CV curves of Zn//Cu asymmetric cells. **d** CE performance of different electrolytes. **e** Voltage distribution profile of Zn//MTP//Cu battery. **f** Rate performances. Cycling tests of the Zn//Zn cells at **g** 0.5 mA cm^−2^ and 0.5 mAh cm^−2^ and **h** 1 mA cm^−2^ and 1 mAh cm^−2^. **i** Comparison of the MTP with other reported hydrogels for Zn//Zn cells

The corrosion resistance of the Zn anode was further evaluated via Tafel polarization curves (Fig. 4a). The MTP electrolyte exhibits the lowest corrosion current density of 0.2046 mA cm^−2^, compared to 0.2625 mA cm^−2^ for PAM and a much higher 1.2745 mA cm^−2^ for ZS. This indicates that the MTP hydrogel reduces corrosion tendency and slows corrosion speed. Consistent with this, the electrochemical stability window (ESW) measured by linear sweep voltammetry (LSV) is expanded to 2.638 V for MTP, compared to 2.418 V for PAM and 2.307 V for ZS (Fig. S13). Notably, both the HER and oxygen evolution reaction (OER) onset potentials were significantly shifted toward more negative and positive potentials, respectively. These results collectively demonstrate that the synergistic MT-PAM network suppresses water-induced corrosion and mitigates electrochemical degradation caused by interfacial side reactions [58]. The deposition mechanism of Zn^2+^ was investigated via chronoamperometry (CA) under a constant overpotential of 150 mV (Fig. 4b). In the ZS electrolyte, the current density continuously increased for the first 107 s, a characteristic signature of uncontrolled 2D planar diffusion that typically leads to dendrite formation [59]. In stark contrast, the current response in the MTP system stabilized rapidly within just 22 s, significantly faster than PAM (56 s), indicating a swift transition to a stable and diffusion-controlled 3D nucleation. This confirms that the MTP electrolyte effectively regulates the Zn^2+^ flux, guiding uniform deposition. The nucleation kinetics were further probed using cyclic voltammetry (CV) on Zn//Cu half-cells (Fig. 4c). The nucleation overpotential (NOP) for the MTP electrolyte was measured at 68 mV, which is 29 and 18 mV higher than that of ZS and PAM, respectively. A higher NOP implies a larger energy barrier for nucleation, which promotes the formation of finer grains and more homogeneous deposition [60]. Additionally, the reversibility of Zn plating/stripping was assessed using Zn//Cu half-cells at 1 mA cm^−2^ and 1 mAh cm^−2^ (Figs. 4d and S14). Cells utilizing ZS and PAM electrolytes failed after only 190 and 385 cycles, respectively. Conversely, the MTP-based cell maintained stable cycling for over 850 cycles with a high average CE of 98.57%. Notably, voltage profiles extracted at various intervals (2nd, 50th, 100th, 600th and 800th cycles) exhibit negligible hysteresis variation (Fig. 4e), demonstrating the exceptional reversibility and long-term durability of the Zn anode enabled by the bioinspired MTP electrolyte.

To evaluate the practical viability of the MTP electrolyte under demanding conditions, rate capability tests were conducted on Zn//Zn symmetric cells at current densities ranging from 0.5 to 8 mA cm^−2^ (Fig. 4f). Cells employing ZS and PAM electrolytes failed rapidly due to short circuit. In stark contrast, the MTP-based cell exhibited robust stability over 10 cycles, demonstrating its superior tolerance to high-rate stripping/plating. Long-term cycling tests further highlighted the exceptional durability of the MTP electrolyte. At 0.5 mA cm^−2^/0.5 mAh cm^−2^, the MTP-based Zn//Zn symmetric cells achieved an ultralong lifespan of 4600 h (over 6 months) with negligible voltage hysteresis (Fig. 4g). In contrast, ZS and PAM-based cells only maintained stable cycling for 240 and 1100 h, respectively. Furthermore, even at a higher current density of 1 mA cm^−2^/1 mAh cm^−2^, the MTP-based cell exhibited stable operation for 2680 h (Fig. 4h). Conversely, the Zn//Zn symmetric cells using ZS and PAM cells failed after only 145 and 329 h, respectively, succumbing to severe dendrite-induced short circuits. Notably, Zn anode utilizing MTP hydrogel electrolyte achieves a superior cumulative capacity of 3200 mAh cm^−2^ at 4 mA cm^−2^/4 mAh cm^−2^, substantially outperforming most recently reported hydrogel electrolytes, including those based on 2D materials (Fig. 4i and Table S3) [16, 61–70], validating the efficacy of the bioinspired hierarchical design.

### Zn Plating Behaviors and Interface Chemistry

**Fig. 5:**
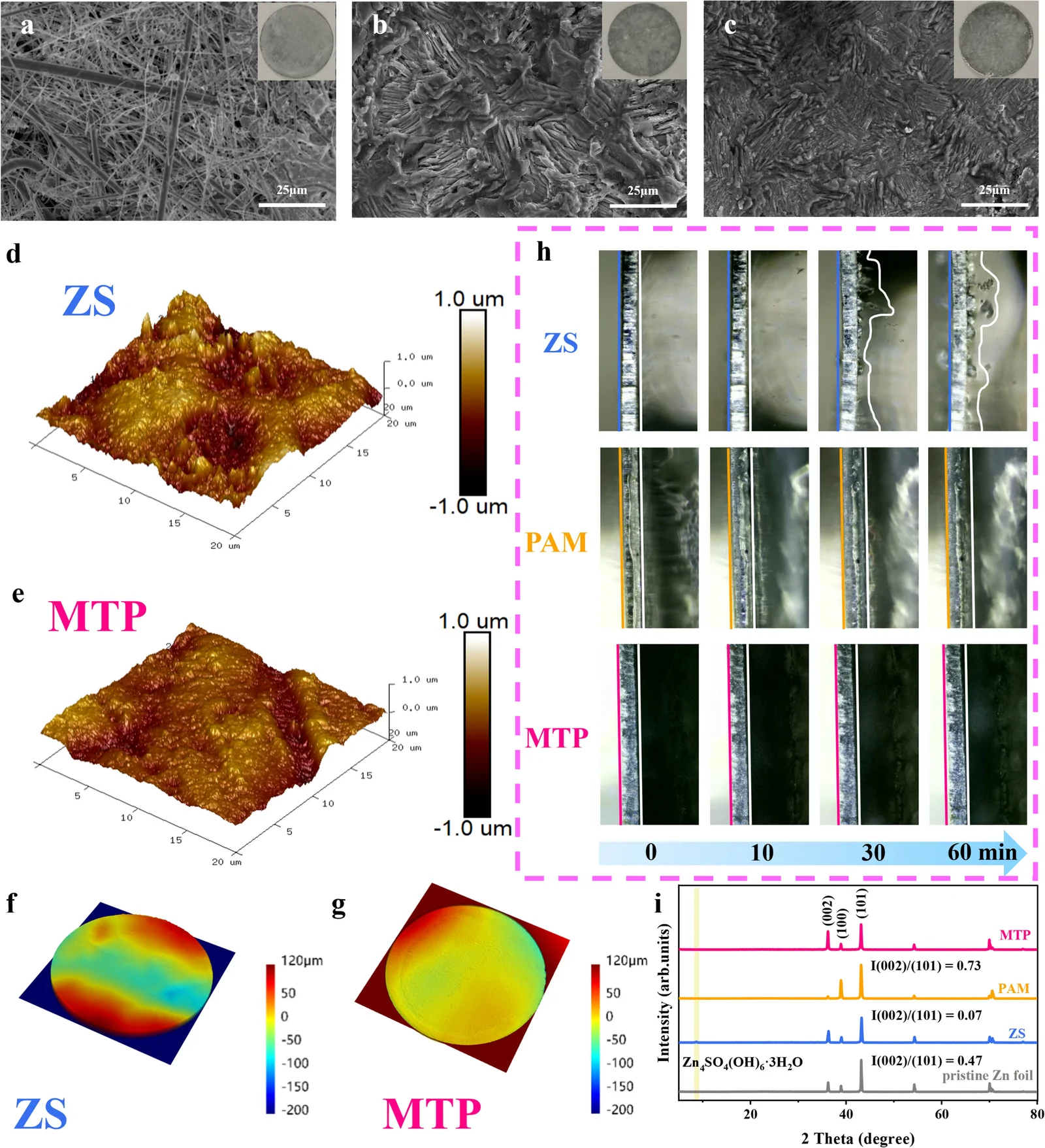
Interfacial characterization of the cycled Zn anode. SEM images of the Zn anodes after 200 cycles at 2 mA cm^−2^ and 2 mAh cm^−2^ in **a** ZS, **b** PAM and **c** MTP. AFM images of Zn sheet after cycling in **d** ZS and **e** MTP. LCSM images of Zn sheet after cycling in **f** ZS and **g** MTP. **h** In situ dendrite observation in various electrolytes. **i** XRD patterns of Zn anodes after cycling for 1 h at 10 mA cm^−2^ and 10 mAh cm^−2^ in ZS, PAM and MTP electrolytes

The suppression of zinc dendrites and regulation of deposition morphology are critical for the long-term stability of ZIBs [71, 72]. To visualize this, Zn anodes cycled at 2 mA cm^−2^/2 mAh cm^−2^ were examined. SEM images (Figs. 5a, S15, and S16) reveal that disordered, needle-like dendrites formed on the anode in ZS electrolyte after just 50 cycles, evolving into a loose and porous structure after 200 cycles. While the PAM electrolyte mitigated this to some extent (Fig. 5b), the anode cycled in MTP displayed a remarkably dense, flat, and dendrite-free morphology with fine grain sizes even after 200 cycles (Fig. 5c). Moreover, this corrosion morphology was quantified by atomic force microscopy (AFM) and laser confocal scanning microscopy (LCSM). After 200 cycles, the anode in ZS exhibited a rough surface with a roughness of 161 nm (Figs. 5d and S17). In contrast, the MTP-cycled anode retained a much smoother surface with a roughness of only 105 nm (Fig. 5e). Full-area LCSM scans confirmed these findings, showing significantly reduced height variations for the MTP system (Fig. 5f, g), where the average surface roughness was reduced from 1.1229 µm (ZS) to 1.0177 µm. Furthermore, in situ optical microscopy captured the real-time evolution of the interface under an aggressive current of 10 mA cm^−2^ (Fig. 5h). Rapid dendrite proliferation was observed in the ZS electrolyte within 30 min and significantly after 60 min due to the tip effect. Conversely, the MTP electrolyte maintained a pristine electrode interface throughout the 60-min test, confirming its ability to regulate Zn^2+^ flux and suppress heterogeneous nucleation.

Subsequently, the crystallographic orientation of the deposited zinc was analyzed by XRD (Fig. 5i). The ZS-cycled anode displayed strong peaks corresponding to the by-product Zn_4_SO_4_(OH)_6_·3H_2_O, which were notably absent in the MTP sample. More importantly, the MTP electrolyte induced a preferred orientation of zinc deposition. The intensity ratio of the (002) to (101) planes (*I*_(002)_/*I*_(101)_) for MTP reached 0.73, significantly higher than that of ZS (0.47) and PAM (0.07). Since the Zn (002) basal plane possesses the lowest surface energy and highest resistance to corrosion and dendrite growth [73, 74], this (002)-textured deposition is a key factor contributing to the superior stability of the MTP-based batteries. In summary, the MTP electrolyte promotes highly oriented (002) deposition and homogenizes Zn^2+^ flux via its bioinspired transport channels, thereby effectively suppressing dendrite growth and ensuring ultralong cycling life.

### Electrochemical Performance of Zn//Z-VO Full Cells

To evaluate the practical viability of the MTP electrolyte in aqueous ZIBs, we synthesized the Zn^2+^-pillared V_2_O_5_·_3_H_2_O (Z-VO) as the cathode material by incorporating ZnSO_4_ into V_2_O_5_. The pre-intercalation of Zn^2+^ expands the interlayer spacing, thereby enhancing structural stability and facilitating ion diffusion [35, 75]. XRD analysis (Fig. S18a) confirms that the Z-VO powder matches the monoclinic V_2_O_5_·_3_H_2_O phase (JCPDS No. 07–0332), with the (001) peak shifting to a lower angle, indicative of successful interlayer expansion. Morphological characterization via SEM and TEM (Fig. S18b-e) reveals a well-defined layered nanosheet structure ideal for reversible Zn^2+^ storage, while energy-dispersive X-ray spectroscopy (EDS) mapping (Fig. S18f-h) confirms the uniform distribution of V, O and Zn elements.

**Fig. 6:**
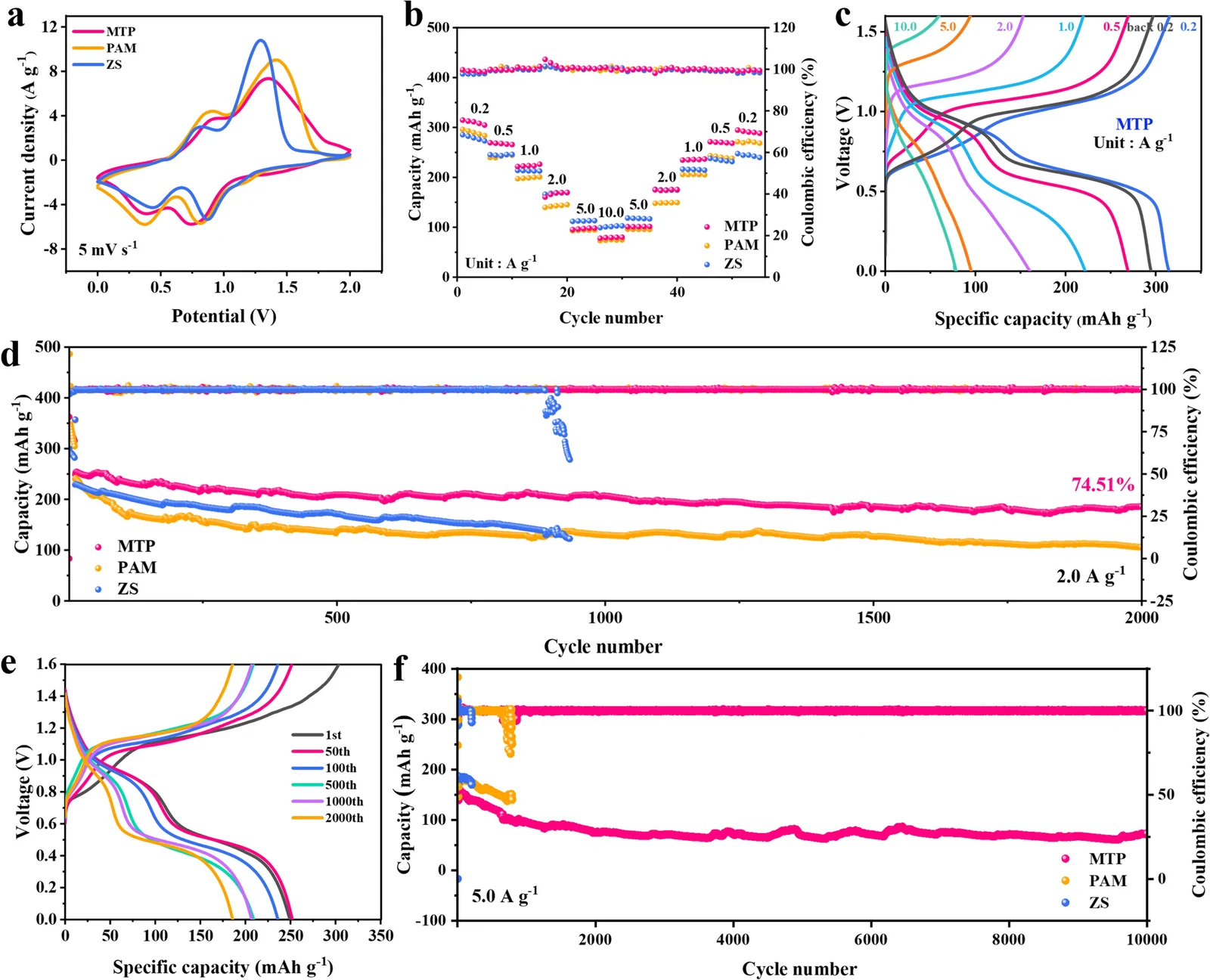
**a** CV curves of Zn//Z-VO full batteries. **b** Rate performances of different electrolytes. **c** GCD curves. Cycling stability of Zn//Z-VO batteries coupled with ZS, PAM and MTP at **d** 2 A g^−1^ and **f** 5 A g^−1^. **e** GCD curves during the long cycle at 2 A g^−1^

The electrochemical performance of the Zn//Z-VO full cells was evaluated via CV (Figs. 6a and S19). Two distinct pairs of redox peaks (1.354/0.744 V and 0.918/0.387 V) were observed, corresponding to the multi-step redox reactions of V^5+^/V^4+^ and V^4+^/V^3+^ accompanying Zn^2+^ intercalation/extraction [76]. LSV measurements revealed that MTP electrolyte broadened the ESW (Fig. S20). This indicates that MTP effectively suppresses side reactions, such as electrolyte oxidation decomposition, thereby enhancing the electrochemical stability and cycle life of the battery. Furthermore, rate capability tests (Fig. 6b, c) demonstrate that the MTP-based full cell delivers superior specific capacities of 314.75, 269.29, 221.90, 169.77, 95.36 and 78.09 mAh g^−1^ at current densities ranging from 0.2 to 10.0 A g^−1^. Crucially, a high capacity of 294.79 mAh g^−1^ was retained upon returning to 0.2 A g^−1^, confirming its excellent reversibility.

Long-term cycling stability of Zn//Z-VO full cells assembled with different electrolytes was assessed at 2 A g^−1^ (Fig. 6d, e). The MTP-based full cell delivered a high specific capacity of 249.41 mAh g^−1^ and maintained with 74.51% retention after 2000 cycles, alongside a near-unity CE (99.98%). In stark contrast, the ZS-based full cell failed catastrophically after 890 cycles due to short circuiting, while the PAM-based cell suffered rapid capacity decay (44.04% retention). Even under a higher current density of 5 A g^−1^, the MTP-based full cell exhibited exceptional durability, cycling stably for 10,000 cycles with a retained capacity of 72.97 mAh g^−1^ (Fig. 6f). Conversely, ZS and PAM-based full cells failed rapidly within 201 and 671 cycles, respectively. This outstanding cycling stability is attributed to the hierarchical MTP network, which accelerates ion transport kinetics and effectively suppresses interfacial side reactions and dendrite growth.

**Fig. 7:**
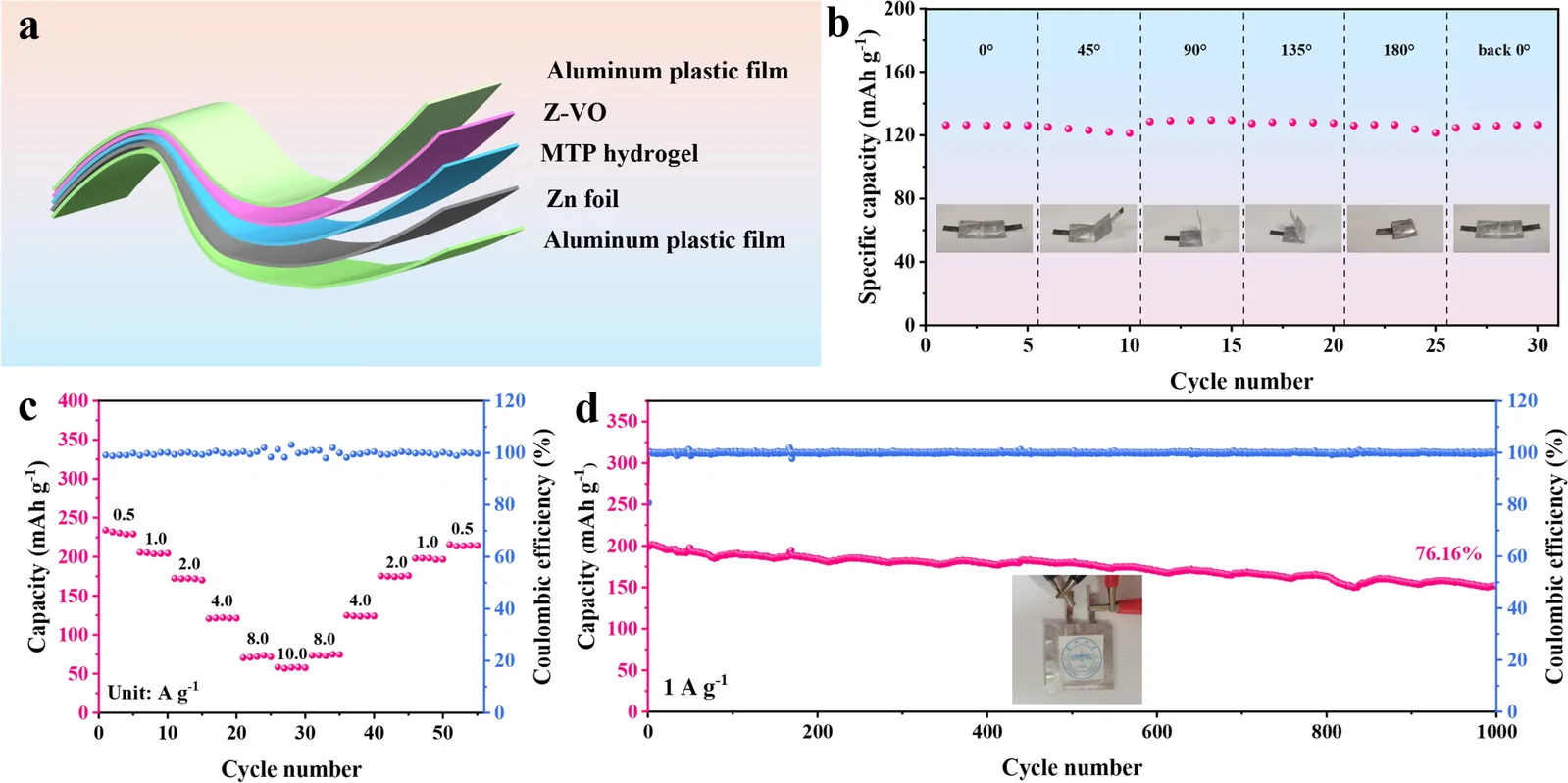
**a** Schematic diagram of flexible Zn//Z-VO pouch cell. **b** Specific capacity of pouch cell at different bending angles. **c** Rate performances of pouch cell ranging from 0.5 to 10 A g^−1^. **d** Long cycling stability of pouch cell at 1 A g^−1^

With the rising demand for wearable electronics, the mechanical robustness of energy storage devices is critical [77, 78]. To demonstrate the practical potential of the MTP electrolyte, flexible Zn//Z-VO pouch cells were assembled (Fig. 7a). The mechanical integrity was tested on a 1 × 3 cm^2^ pouch cell under various bending conditions (Fig. 7b). The pouch cell delivered stable specific capacities (~ 126 mAh g^−1^ at 5 A g^−1^) regardless of the bending angle (0°, 45°, 90°, 135°, 180°), with minimal capacity fluctuation upon returning to the flat state, confirming excellent electrode–electrolyte contact under deformation. To further validate scalability, a larger 2 × 2 cm^2^ pouch cell was fabricated. This device achieved an ultra-high specific capacity of 234.22 mAh g^−1^ at 0.5 A g^−1^ (Fig. 7c) and demonstrated robust cycling stability at 1 A g^−1^, retaining 76.16% of its initial capacity (200.81 mAh g^−1^) after 1000 cycles (Fig. 7d). These results compellingly suggest that the bioinspired MTP electrolyte is a promising candidate for high-performance, flexible, and safe energy storage systems.

## Conclusion

In summary, we have successfully engineered a bioinspired hierarchical hydrogel electrolyte (MTP) that mimics the “adhesion-conduction” architecture of spider webs. By incorporating TA-modified MXene nanosheets into a PAM skeleton, this design resolves the intrinsic trade-off between mechanical robustness and ionic conductivity. Mechanistically, the PAM network ensures structural integrity, while the abundant polar groups on MXene and phenolic hydroxyls on TA act as “sticky sites”. These sites significantly accelerate Zn^2+^ desolvation kinetics and construct directed 3D transport channels, endowing the MTP hydrogel with a remarkable ionic conductivity of 27.69 mS cm^−1^ and a high Zn^2+^ transference number of 0.833. Enabled by this synergistic design, the MTP electrolyte demonstrates exceptional electrochemical stability. Zn//Zn symmetric cells achieve an ultralong lifespan of 4600 h (0.5 mA cm^−2^, 0.5 mAh cm^−2^) and maintain stability even under harsh conditions (800 h at 4 mA cm^−2^). Furthermore, Zn//Z-VO full cells exhibit outstanding durability, achieving cycling stably for 2000 cycles at 2 A g^−1^ and 10,000 cycles at 5 A g^−1^. Notably, the practical viability of this system is validated by large-area pouch cells, which deliver an ultra-high specific capacity of 234.22 mAh g^−1^. This work not only provides a high-performance electrolyte for flexible ZIBs but also offers a pioneering bioinspired strategy to regulate ion transport kinetics for stable metal anodes.

## Supplementary Information

Below is the link to the electronic supplementary material.

Supplementary file1 (DOCX 8.41 MB)
