# Supplementary material for: Bioinspired Hierarchical Hydrogel Electrolyte for Ultralong-Life Flexible Zinc-Ion Batteries
Source: Nanomicro Lett. 2026 Jun 10;18:399. doi: 10.1007/s40820-026-02246-0 (PMC13249997; doi:10.1007/s40820-026-02246-0)
Supplement: Supplementary file 1 — Supplementary file1 (DOCX 8.41 MB) [file 40820_2026_2246_MOESM1_ESM.docx]

Supporting Information for

**Bioinspired Hierarchical Hydrogel Electrolyte for Ultralong-Life Flexible Zinc-Ion Batteries**

Ran Wang^1^, Qian Gao^1^, Runhai Wu^1^, Yongqi Mi^1^, Shaopei Yang^1^, Hongxiao Wang^1^, Ting Wan^1^, Sehrish Gull^2^, Kefeng Xie^3,^*, Guankui Long^2,^*, Pengcheng Du^1,^*

^1^State Key Laboratory of Natural Product Chemistry and Institute of Polymer Science and Engineering, College of Chemistry and Chemical Engineering, Lanzhou University, Lanzhou 730000, P. R. China

^2^Frontiers Science Center for New Organic Matter, Tianjin Key Lab for Rare Earth Materials and Applications, Renewable Energy Conversion and Storage Center (RECAST), School of Materials Science and Engineering, Nankai University, Nankai University, Tianjin 300350, P. R. China

^3^School of Chemistry and Chemical Engineering, Lanzhou Jiaotong University, Lanzhou 730070, P. R. China

*Corresponding authors. E-mail: xiekefeng@mail.lzjtu.cn (Kefeng Xie); longgk09@nankai.edu.cn (Guankui Long); dupch@lzu.edu.cn (Pengcheng Du)

**S1 Experimental section**

**S1.1 Characterizations**

The crystallographic structures were analyzed by X-ray diffraction (XRD) using a Shimadzu XRD-6000 diffractometer. Morphologies of the Zn anodes, hydrogels, and Z-VO cathodes were characterized using a scanning electron microscope (SEM, SU8600). The microstructure was further examined by transmission electron microscopy (TEM, JEM-2100). Surface roughness was quantified using an atomic force microscope (AFM, Dimension Icon) and a laser confocal scanning microscope (LCSM, KC-X3000). Chemical compositions and interactions were investigated using Fourier transform infrared spectroscopy (FTIR, Nicolet iS5) and X-ray photoelectron spectroscopy (XPS, PHI-5702). Mechanical properties, including tensile strength (5×1 cm^2^ samples) and adhesion strength (2×2 cm^2^ samples), were evaluated using a universal testing machine (AGX-V 500N) at room temperature. Adhesive performance was evaluated by measuring the separation force of zinc foils bonded to a hydrogel at a tensile rate of 100 mm min^-1^.

**S1.2 Electrochemical Measurements**

Electrochemical performances of Zn//Zn symmetric batteries, Zn//Cu half batteries and Zn//Z-VO full batteries were evaluated using CR-2032 coin-type cells under room temperature in ambient air (~20 ℃). Zn foils (diameter: 10 mm, thickness: 0.1 mm) and Cu foils (diameter: 12 mm) were used as electrodes. For cells using aqueous ZS electrolyte, a glass fiber separator was employed, whereas hydrogel electrolytes served as both the electrolyte and the separator. Long-term cycling and rate capability tests were conducted on a LANHE-CT3004A battery testing system. Coulombic efficiency (CE) was evaluated in Zn//Cu half-cells at 1 mA cm^-2^ with a fixed capacity of 1 mAh cm^-2^. Cyclic voltammetry (CV), linear sweep voltammetry (LSV), chronoamperometry (CA), and electrochemical impedance spectroscopy (EIS) were performed on a CHI660E electrochemical workstation.

Tafel plots were recorded in Zn//Zn symmetric cells from -0.3 V to 0.3 V at a scan rate of 1 mV s^-1^. The CA test was setting a bias voltage of 150 mV for 300 s. Ionic conductivity ($\sigma$) was measured using EIS in a blocking Ti//Electrolyte//Ti configuration and calculated using the equation:

$$\begin{aligned} \sigma=\frac{L}{RA}\#\left( \text{S}\text{1} \right) \end{aligned}$$

where σ (S cm^-1^) is the ionic conductivity, L (cm) is the hydrogel thickness, A (cm^2^) is the contact area, and R (Ω) is the bulk resistance (intercept on the Z’ axis).

Nucleation overpotential was tested in Zn//Cu cells from -0.2 V to 0.5 V at 5 mV s^-1^. LSV determined electrochemical stability window (ESW) in Zn//Ti cells from -1 V to 3 V at 0.5 mV s^-1^. Zn^2+^ transference number ($\text{t}_{\text{Zn}^{\text{2+}}}$) was determined via the Bruce-Vincent method in a Zn//Zn symmetric cell. This involved a chronoamperometry step (5 mV polarization for 1000 s) combined with EIS measurements before and after polarization. $\text{t}_{\text{Zn}^{\text{2+}}}$was calculated as:

$$\begin{aligned} t_{{Zn}^{2+}}=\frac{I_{s}\left( \Delta V-I_{0}R_{0} \right)}{I_{0}\left( \Delta V-I_{s}R_{s} \right)}\#\left( \text{S}\text{2} \right) \end{aligned}$$

Where $\Delta V$ is the bias voltage, *R_o_* and *R_s_* are the initial and steady-state charge transfer resistances of the electrode, and *I_o_* and *I_s_* are the initial and steady-state currents, respectively.

**S2 Computational methods**

Density Functional Theory (DFT) calculations were performed using Gaussian 16 software. Geometry optimizations and frequency analyses were conducted at the ωB97X level of theory, employing the D3BJ dispersion correction to account for weak interactions. No imaginary frequencies were found, confirming that the optimized structures correspond to local minima. The 6-31G** basis set was used for C, H, O, and N atoms, while the Stuttgart pseudopotential and basis set were adopted for Zn [S1].

The binding energy (*E_binding_*) between components was calculated as:

$$\begin{aligned} E_{binding}=E_{A+B}-E_{A}-E_{B}\#\left( \text{S}\text{3} \right) \end{aligned}$$

where *E_A+B_* is the energy of the system combined with two complexes, *E_A_* and *E_B_* are the energies of complex A and complex B, respectively.

The energy (*ΔE*) of the desolvation process is defined as:

$$\begin{aligned} \Delta E=E_{{x-Zn\left[ 4\left( H_{2}O \right) \right]}^{2+}}-E_{{x-Zn\left[ 5\left( H_{2}O \right) \right]}^{2+}}+E_{H_{2}O}\#\left( \text{S}\text{4} \right) \end{aligned}$$

Where *x* is H_2_O, PAM and TA molecule, $E_{H_{2}O}$ corresponds to the energy of an isolated water molecule in vacuum.

**S3 Supplementary Figures and Tables**

**
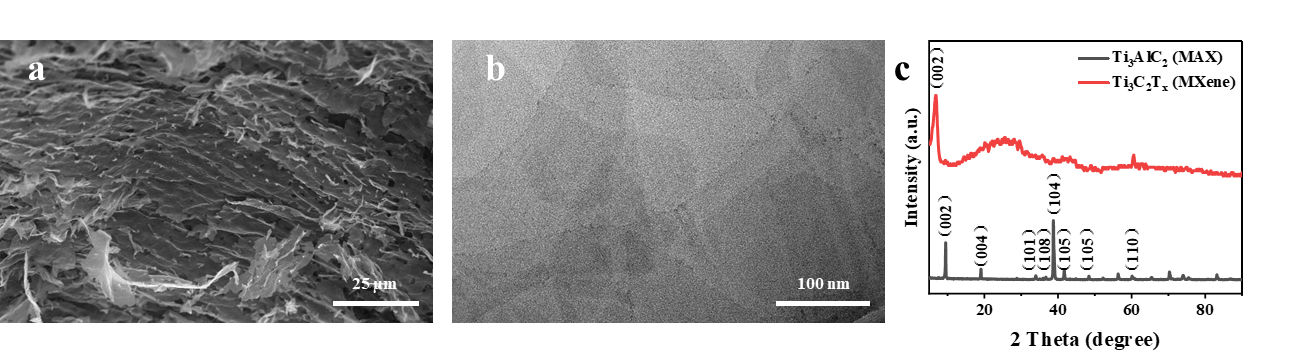
**

**Fig. S1** (a) SEM image of MXene. (b) TEM image of MXene. (c) XRD pattern of Ti_3_AlC_2_ and Ti_3_C_2_T_x_ (MXene).

XRD analysis reveals that the (002) diffraction peak shifts from 9.52° to 6.88°, which is attributed to the expansion of interlayer spacing caused by the intercalation of F^-^ and Li⁺. This result confirms the successful synthesis of MXene nanosheets.

**
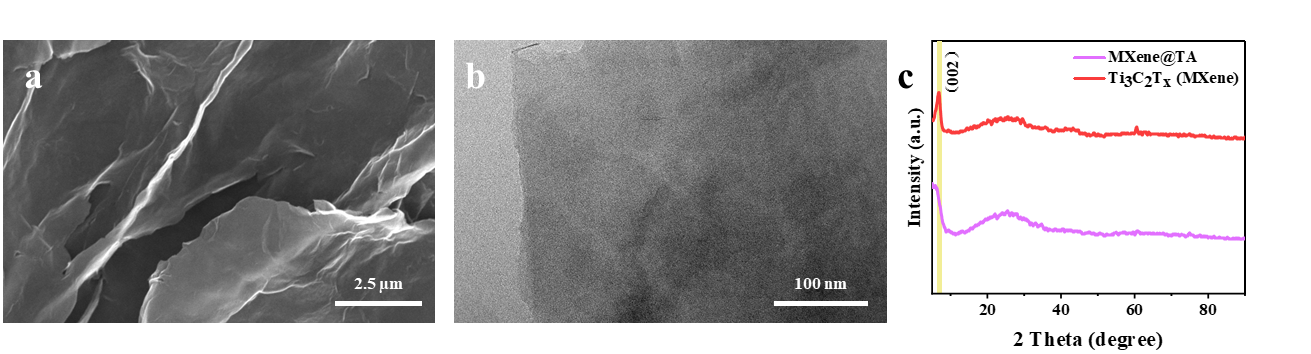
**

**Fig. S2** (a) SEM image of MXene@TA. (b)TEM image of MXene@TA. (c) XRD pattern of MXene and MXene@TA.

XRD pattern reveals a leftward shift in the (002) diffraction peak after the introduction of TA, indicating the successful intercalation of TA molecules between MXene interlayers.

**Fig. S3** XPS spectra of Mxene and Mxene@TA.

**
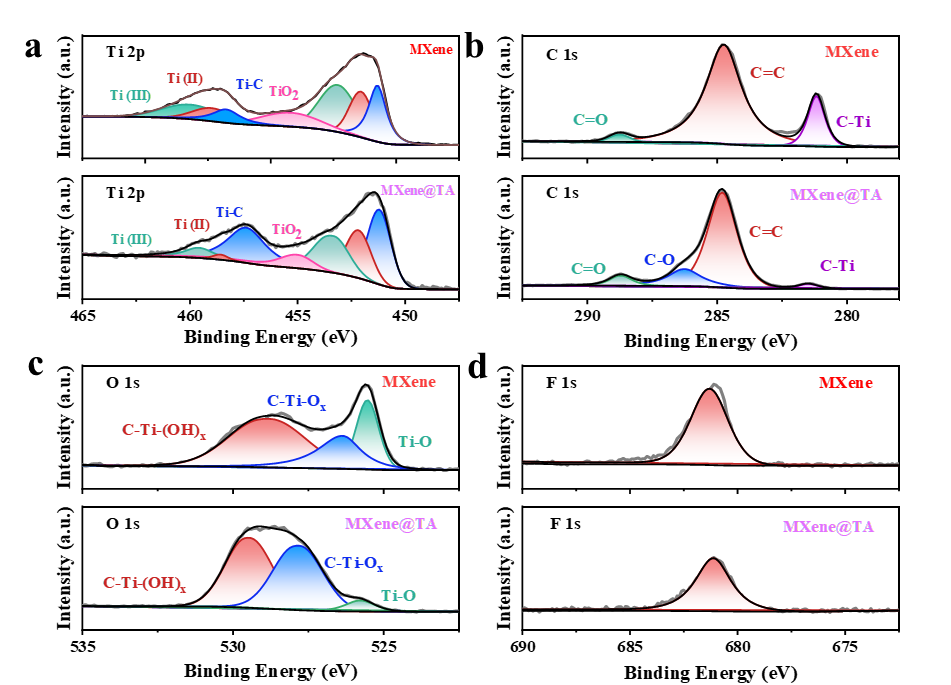
**

**Fig. S4** XPS spectra of (a) Ti 2p, (b) C 1s, (c) O 1s and (d) F 1s for MXene and MXene@TA.

In the C 1s spectrum, compared with MXene, the content of C=O bonds in MT increases significantly, and an additional C-O bond with a relative content of 11.60% is observed, originating from the oxygen-rich functional groups of TA. The O 1s spectrum shows that the peak position of C-Ti-O_x_ shifted from 528.79 eV to 527.80 eV, which is attributed to the formation of hydrogen bonds between MXene and TA, further confirming the successful synthesis of MT.

**
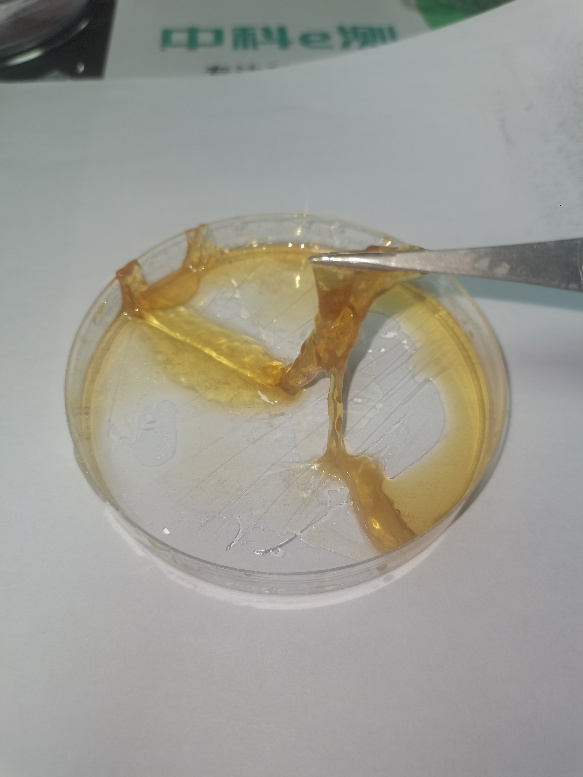
**

**Fig. S5** Image of TP hydrogel.


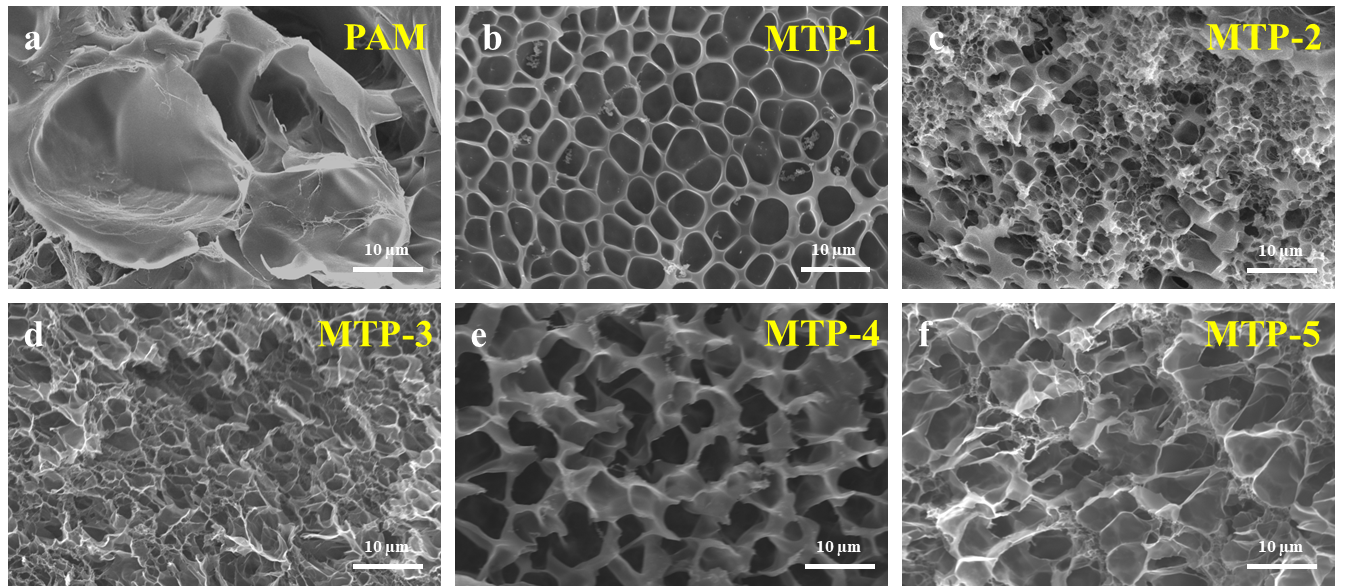


**Fig. S6** SEM images of (a) PAM and (b-f) MTP series hydrogels.

**Fig. S7** Water retention of MTP and PAM hydrogels at 25 ℃.

**
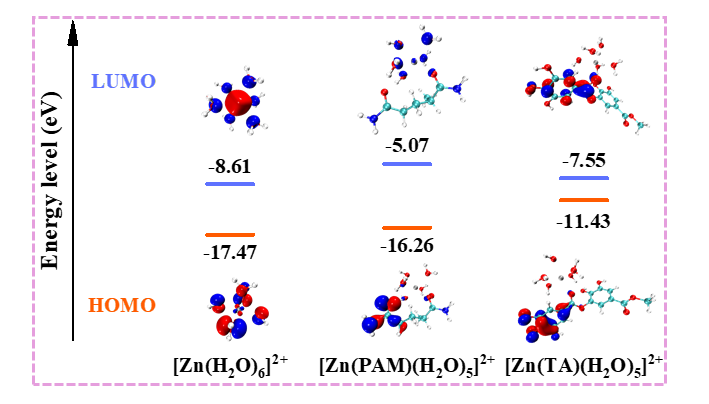
**

**Fig. S8** HOMO-LUMO energy level distribution of [Zn(H_2_O)_6_]^2+^, [Zn(PAM)(H_2_O)_5_]^2+^, and [Zn(TA)(H_2_O)_5_]^2+^.

**Fig. S9** Cycling tests of the Zn//Zn-symmetric battery coupled with different electrolytes under 4 mA cm^-2^, 4 mAh cm^-2^.


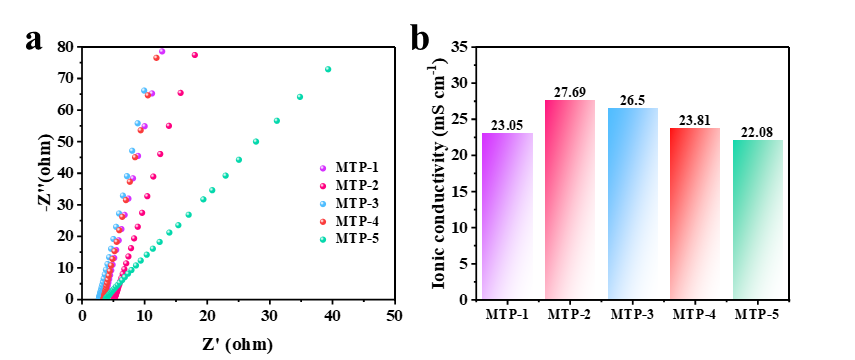


**Fig. S10** Ionic conductivity of MTP series electrolytes.

**Fig. S11** Ionic conductivity of diverse electrolytes.


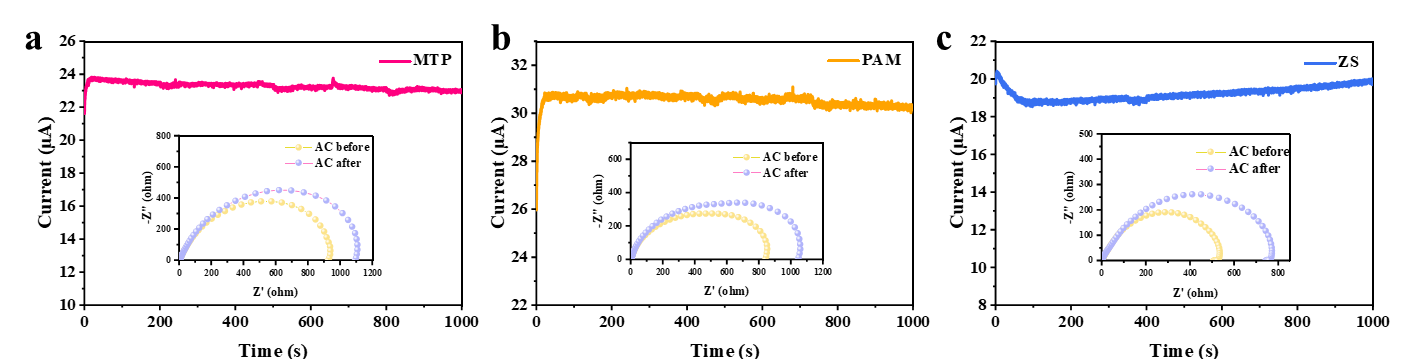


**Fig. S12** The Zn^2+^ transference number in (a) MTP gel electrolyte, (b) PAM gel electrolyte, and (c) ZS electrolyte.

**Table S1** Comparison of ionic conductivity and Zn^2+^ transference number among MTP and recently reported works.

| Strategies | Ionic conductivity (mS cm^-1^) | Zn^2+^ transference | Refs. |
| --- | --- | --- | --- |
| **MTP** | **27.69** | **0.833** | **This work** |
| PCG20-PC5 | 16.18 | 0.45 | [S2] |
| PAHE352 | 23.10 | 0.69 | [S3] |
| PAP | 25.4 | 0.70 | [S4] |
| Phos-XK-1 | 20.72 | 0.83 | [S5] |
| PICZ | 25.62 | 0.76 | [S6] |
| P-MCs | 22.02 | 0.712 | [S7] |
| APHE | 14.1 | 0.72 | [S8] |
| IL-Br | 0.739 | 0.61 | [S9] |
| HE-RS | 3.9 | 0.40 | [S10] |
| Gel/SA-acetate | 13.6 | 0.68 | [S11] |

**Fig. S13** LSV curves Zn//Zn symmetric cells using different electrolytes.

**Fig. S14** CE test curves of different electrolytes.

**Table S2** Cumulative capacity comparison of Zn//Zn symmetric cells using MTP gel electrolyte at high current density with previous reports

| Strategies | Current  density  (mA cm^-2^) | Lifespan  (h) | Cumulative  Capacity  (mAh cm^-2^) | Ref. |
| --- | --- | --- | --- | --- |
| **MTP** | **0.5**  **1**  **4** | **4600**  **2680**  **800** | **2300**  **2680**  **3200** | **This work** |
| IL-Br | 0.2 | 2400 | 480 | [S9] |
| USPH-5 | 0.5 | 1000 | 500 | [S12] |
| ZGPE | 0.5 | 3600 | 1800 | [S13] |
| CZHE | 1 | 1200 | 1200 | [S14] |
| PAMS30-ZH | 1 | 1700 | 1700 | [S15] |
| PMPC | 1 | 3300 | 3300 | [S16] |
| PASHE | 2 | 400 | 800 | [S17] |
| In-situ GPE | 3 | 200 | 600 | [S18] |
| PDMAPS | 3 | 800 | 2400 | [S19] |
| PC-PVA/Zn(CF_3_SO_3_)_2_ | 4 | 400 | 1600 | [S20] |
| PSC-gel | 5 | 500 | 2500 | [S21] |

**Table S3** Comparison of integrated performance among MTP and 2D material-based hydrogel electrolytes

| Strategies | Current  density  (mA cm^-2^) | Lifespan  (h) | Cumulative  Capacity  (mAh cm^-2^) | Ionic conductivity  (mS cm^-1^) | Elongation at Break  (%) | Refs. |
| --- | --- | --- | --- | --- | --- | --- |
| **MTP** | **0.5**  **1**  **4** | **4600**  **2680**  **800** | **2300**  **2680**  **3200** | **27.69** | **377** | **This work** |
| MXene-CNF | 5 | 550 | 2750 | 0.52 | 24 | [S22] |
| MXene-PAM-PVA | 1 | 1900 | 1900 | 17.36 | 295.75 | [S23] |
| MMT-PAM | 0.5 | 300 | 150 | 20.7 | 1075 | [S24] |
| PCG20-PC5 | 1 | 2200 | 2200 | 16.18 | 320 | [S2] |
| P-MCs | 5 | 600 | 3000 | 22.02 | 2400 | [S7] |

**
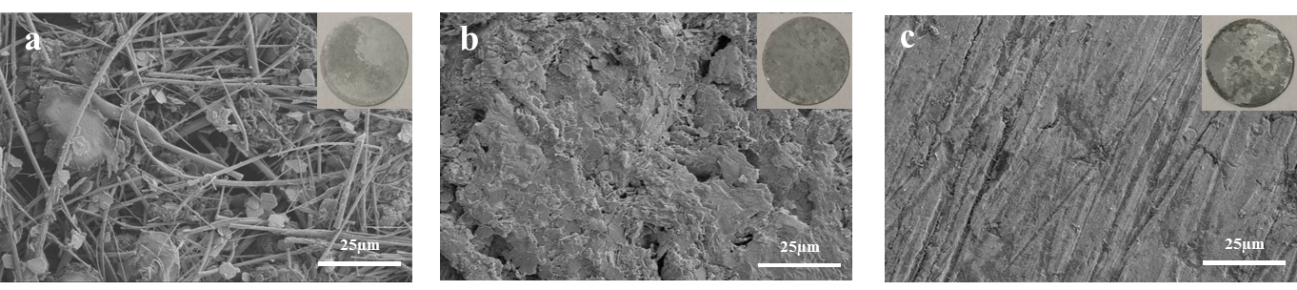
**

**Fig. S15** SEM images of the Zn anodes after 50 cycles at 2 mA cm^-2^, 2 mAh cm^-2^ in (a) ZS, (b) PAM and (c) MTP.


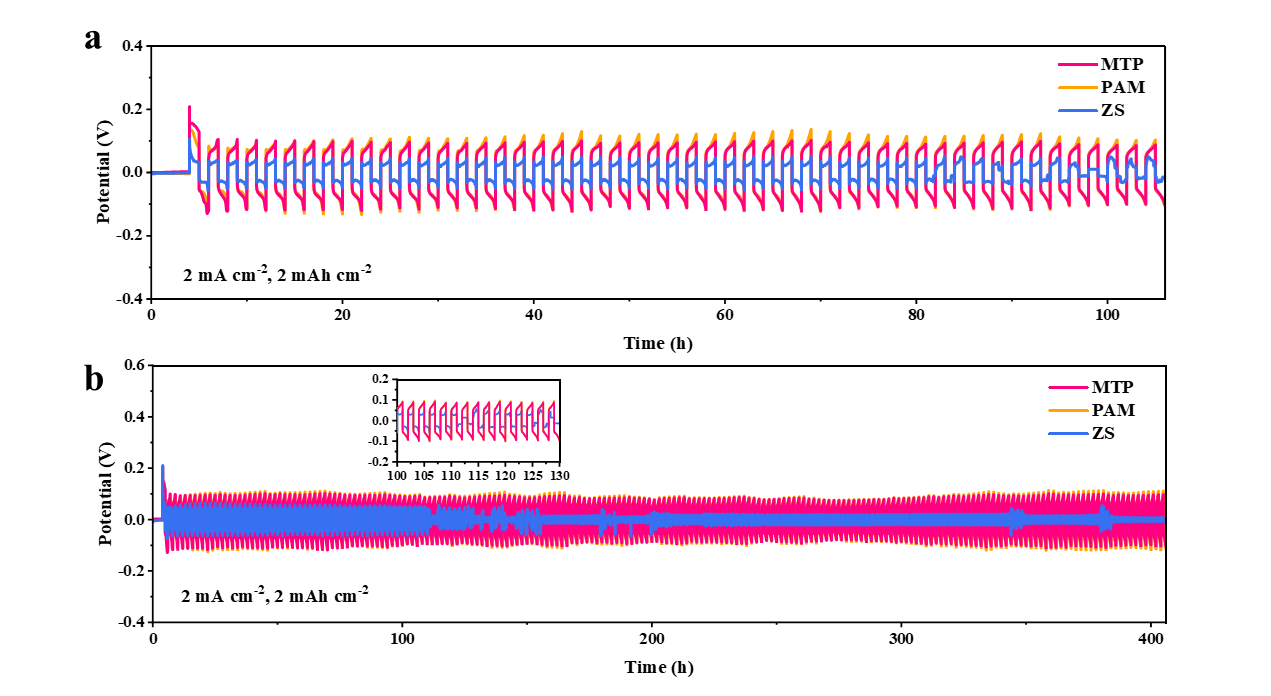


**Fig. S16** Cycling test curves of Zn//Zn symmetric cells assembled with different electrolytes in (a) 50 cycles and (b) 200 cycles.


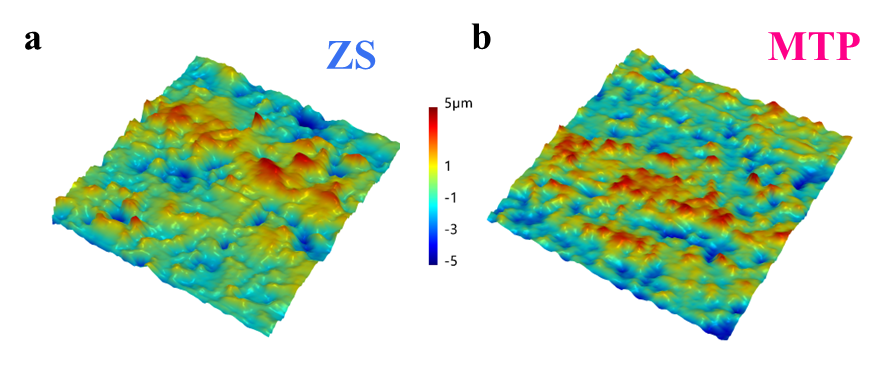
**Fig. S17** LCSM images of the Zn anodes after 200 cycles at 2 mA cm^-2^, 2 mAh cm^-2^ in (a) ZS and (b) MTP.


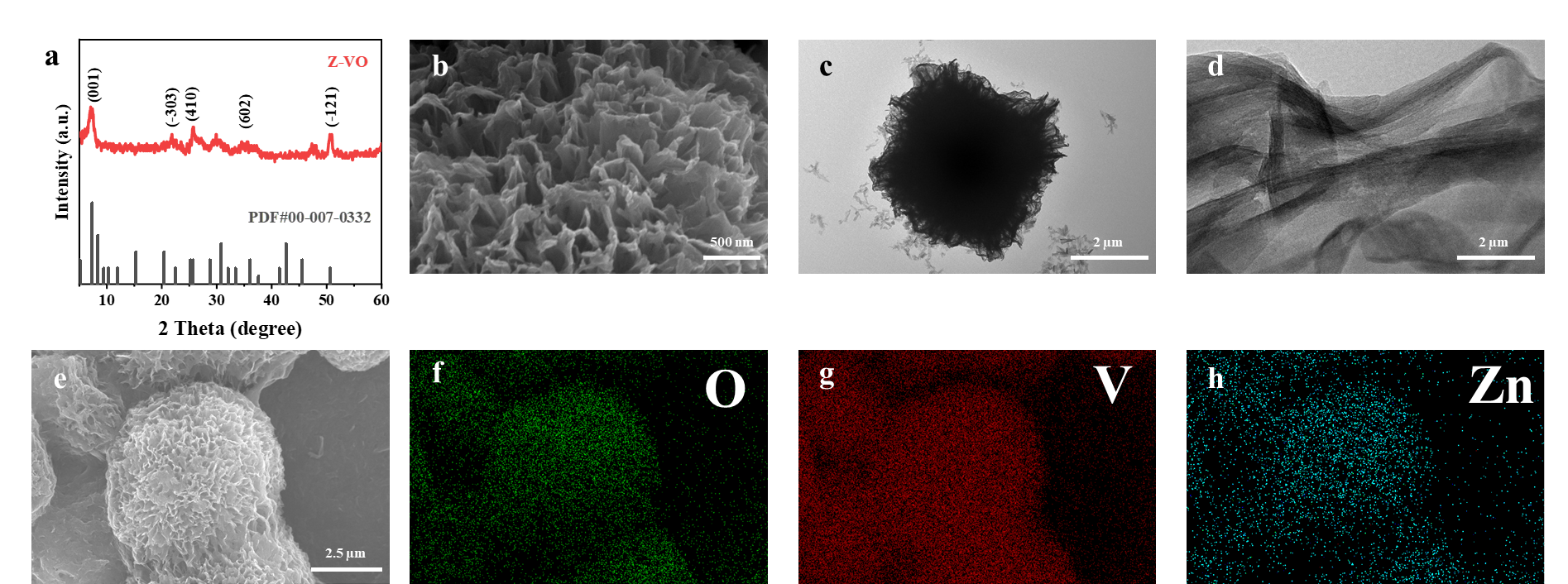


**Fig. S18** (a) XRD pattern of Z-VO. (b) SEM image of Z-VO. (c and d) TEM of Z-VO. (e) SEM image of Z-VO and the corresponding element mapping of (f) O, (g) V and (h) Zn.


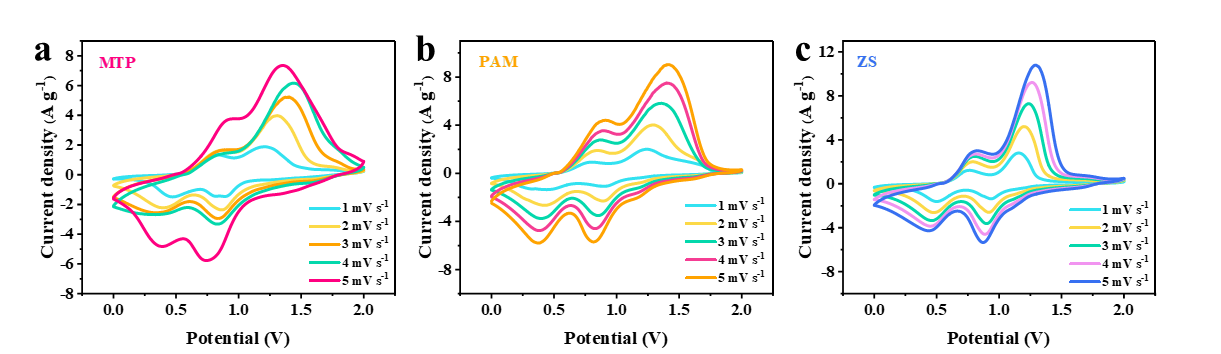


**Fig. S19** CV curves at different scan rates of Zn//Z-VO full cells assembled with (a) MTP, (b) PAM, and (c) ZS.

**Fig. S20** LSV curves of the full cells assembled with different electrolytes.

**Supplementary References**

1. P.C. Hariharan, J.A. Pople, Accuracy of AH*_n_* equilibrium geometries by single determinant molecular orbital theory. Mol. Phys. **27**(1), 209–214 (1974). <https://doi.org/10.1080/00268977400100171>
2. Q. Wang, J. Huang, L. Qi, M. Li, S. Wang et al., A bioinspired gradient hydrogel electrolyte network with optimized interfacial chemistry toward robust aqueous zinc-ion batteries. ACS Nano **19**(29), 26770–26781 (2025). <https://doi.org/10.1021/acsnano.5c06914>
3. S. Qin, R. Qi, Y. Wang, Y. Hu, M. Ma et al., A jointly triggered H_2_ evolution model modulated polyanionic hydrogel electrolyte for reversible Zn chemistry. Adv. Funct. Mater. **35**(35), 2505946 (2025). <https://doi.org/10.1002/adfm.202505946>
4. J. Zhong, C. Xia, T. Zhang, H. Wang, H. Tao et al., Non-covalent molecular engineering of hydrogel electrolytes *via* π-anion confinement and hydrogen-bond reconfiguration for wide-temperature and ultra-stable Zn-ion batteries. Adv. Funct. Mater. **36**(28), e27894 (2026). <https://doi.org/10.1002/adfm.202527894>
5. L. Gou, L. Zhu, W. Wang, Z. Yang, S.-Y. Wang et al., From physical cross-linking to tailored phosphorylation: unlocking high-performance and biocompatible xanthan-konjac hydrogels for zinc-ion batteries. Adv. Mater. **37**(34), 2505132 (2025). <https://doi.org/10.1002/adma.202505132>
6. M. Xu, F. Liu, L. Chen, Y. Lei, Z. Liu et al., Zwitterionic poly(ionic liquid) hydrogel electrolytes with high-speed ion conduction channels for dendrite-free, long-enduring zinc-ion batteries and flexible electronics. Energy Storage Mater. **80**, 104373 (2025). <https://doi.org/10.1016/j.ensm.2025.104373>
7. F. Luo, S. Yang, Q. Wu, Y. Li, J. Zhang et al., Hydrogel electrolytes with an electron/ion dual regulation mechanism for highly reversible flexible zinc batteries. Energy Environ. Sci. **17**(22), 8570–8581 (2024). <https://doi.org/10.1039/D4EE03067B>
8. T. Su, W. Ren, M. Xu, K. Li, T.-Y. Yang et al., A reusable biomass electrolyte with dual-interface regulation towards flexible and durable zinc-iodine batteries. Energy Storage Mater. **78**, 104279 (2025). <https://doi.org/10.1016/j.ensm.2025.104279>
9. Z. Hu, Z. Han, H. Liu, X. Jiang, K. Bai et al., Mechanically strong and tough ionic liquid gel electrolyte for four-electron zinc-iodine batteries. J. Am. Chem. Soc. **147**(50), 46632–46641 (2025). <https://doi.org/10.1021/jacs.5c18431>
10. H. Tian, M. Yao, Y. Guo, Z. Wang, D. Xu et al., Hydrogel electrolyte with regulated water activity and hydrogen bond network for ultra-stable zinc electrode. Adv. Energy Mater. **15**(9), 2403683 (2025). <https://doi.org/10.1002/aenm.202403683>
11. C. Tian, J. Wang, R. Sun, T. Ali, H. Wang et al., Improved interfacial ion migration and deposition through the chain-liquid synergistic effect by a carboxylated hydrogel electrolyte for stable zinc metal anodes. Angew. Chem. Int. Ed. **62**(42), e202310970 (2023). <https://doi.org/10.1002/anie.202310970>
12. Y. Wang, W. Yan, X. Zhu, J. Li, Z. Li et al., Boosting performance of quasi-solid-state zinc ion batteries *via* zincophilic solubilization. Angew. Chem. Int. Ed. **64**(35), e202508556 (2025). <https://doi.org/10.1002/anie.202508556>
13. S. Lee, I.K. Han, N.G. Jeon, Y. Lee, H. Bin Son et al., Promoting homogeneous zinc-ion transfer through preferential ion coordination effect in gel electrolyte for stable zinc metal batteries. Adv. Sci. **10**(34), 2304915 (2023). <https://doi.org/10.1002/advs.202304915>
14. C. Wang, Z. Gong, J.A. Yuwono, Q. Meng, Y. Lyu et al., Ligand-channel-induced ion liberation in crowded zwitterionic hydrogel electrolyte for efficient zinc metal batteries. Nat. Commun. **16**, 11069 (2025). <https://doi.org/10.1038/s41467-025-66041-y>
15. G. Liu, S. Zhang, R. Wang, H. Zeng, R. Liu et al., Inhibiting proton corrosion and hydrogen evolution reaction on the surface of zinc anodes by hierarchical structure hydrogel to realize long-life aqueous zinc metal batteries. Adv. Energy Mater. **16**(1), e03823 (2026). <https://doi.org/10.1002/aenm.202503823>
16. Y. Lei, F. Liu, L. Chen, M. Xu, Y. Hu et al., Polyanionic hydrogel electrolytes to regulate ion transport behavior in long cycle life zinc-ion batteries. Nano Energy **143**, 111284 (2025). <https://doi.org/10.1016/j.nanoen.2025.111284>
17. W. Zhang, F. Guo, H. Mi, Z.-S. Wu, C. Ji et al., Kinetics-boosted effect enabled by zwitterionic hydrogel electrolyte for highly reversible zinc anode in zinc-ion hybrid micro-supercapacitors. Adv. Energy Mater. **12**(40), 2202219 (2022). <https://doi.org/10.1002/aenm.202202219>
18. Y. Yang, Q. He, C. Hu, X. Xie, S. Liang et al., Electron-initiated self-growth *in situ* hydrogel electrolyte with gradient protection interface enables stable zinc metal batteries. ACS Nano **19**(23), 21717–21728 (2025). <https://doi.org/10.1021/acsnano.5c04942>
19. S. Zhang, H. Ao, J. Dong, D. Wang, C. Wang et al., Dipole moment dictates the preferential immobilization in gel electrolytes for Ah-level aqueous zinc-metal batteries. Angew. Chem. Int. Ed. **64**(2), e202414702 (2025). <https://doi.org/10.1002/anie.202414702>
20. Y. Xiong, H. Cheng, Y. Jiang, Z. Fan, X. Li et al., A novel water-reducer-based hydrogel electrolyte for robust and flexible Zn-I_2_ battery. Energy Storage Mater. **74**, 103981 (2025). <https://doi.org/10.1016/j.ensm.2024.103981>
21. Z. Wang, R. Xue, H. Zhang, Y. Zhang, X. Tang et al., A hydrogel electrolyte toward a flexible zinc-ion battery and multifunctional health monitoring electronics. ACS Nano **18**(10), 7596–7609 (2024). <https://doi.org/10.1021/acsnano.4c00085>
22. M. Liu, L. Zhang, J. Rostami, T. Zhang, K. Matthews et al., Tough MXene-cellulose nanofibril ionotronic dual-network hydrogel films for stable zinc anodes. ACS Nano **19**(13), 13399–13413 (2025). <https://doi.org/10.1021/acsnano.5c01497>
23. Z. Xu, L. Mei, W. Ouyang, Z. Pan, C. Wang et al., MXene-reinforced interpenetrating network hydrogel for dendrite-free zinc anodes. ACS Appl. Polym. Mater. **7**(22), 15542–15552 (2025). <https://doi.org/10.1021/acsapm.5c03279>
24. S. Ji, J. Qin, S. Yang, P. Shen, Y. Hu et al., A mechanically durable hybrid hydrogel electrolyte developed by controllable accelerated polymerization mechanism towards reliable aqueous zinc-ion battery. Energy Storage Mater. **55**, 236–243 (2023). <https://doi.org/10.1016/j.ensm.2022.11.050>
